# Effect of an Herbal-Based Injection on 28-Day Mortality in Patients With Sepsis: The EXIT-SEP Randomized Clinical Trial

**DOI:** 10.1001/jamainternmed.2023.0780

**Published:** 2023-05-01

**Authors:** Songqiao Liu, Chen Yao, Jianfeng Xie, Hong Liu, Hongliang Wang, Zhaofen Lin, Bingyu Qin, Difen Wang, Weihua Lu, Xiaochun Ma, Yan Liu, Ling Liu, Chi Zhang, Lei Xu, Ruiqiang Zheng, Feihu Zhou, Zhongmin Liu, Guoqiang Zhang, Lixin Zhou, Jian Liu, Aihua Fei, Guoxiu Zhang, Yimin Zhu, Kejian Qian, Ruilan Wang, Yafeng Liang, Meili Duan, Dawei Wu, Rongqing Sun, Ying Wang, Xijing Zhang, Quan Cao, Mingshi Yang, Minggen Jin, Yan Song, Ling Huang, Fachun Zhou, Dechang Chen, Qun Liang, Chuanyun Qian, Zhongzhi Tang, Zhong Zhang, Qiming Feng, Zhiyong Peng, Renhua Sun, Zhenju Song, Yunbo Sun, Yanfen Chai, Lihua Zhou, Chen Cheng, Li Li, Xiaoyan Yan, Junhua Zhang, Yingzi Huang, Fengmei Guo, Chuan Li, Yi Yang, Hongcai Shang, Haibo Qiu

**Affiliations:** 1 Jiangsu Provincial Key Laboratory of Critical Care Medicine, Department of Critical Care Medicine, Zhongda Hospital, School of Medicine, Southeast University, Nanjing, Jiangsu, China; 2 Peking University Clinical Research Institute, Peking University First Hospital, Beijing, China; 3 Department of Critical Care Medicine, The First Hospital of Shanxi Medical University, Taiyuan, Shanxi, China; 4 Department of Critical Care Medicine, The Second Affiliated Hospital of Harbin Medical University, Harbin, Heilongjiang, China; 5 Department of Emergency and Critical Care, Shanghai Changzheng Hospital, Shanghai, China; 6 Department of Critical Care Medicine, Henan Provincial People’s Hospital, Zhengzhou, Henan, China; 7 Department of Critical Care Medicine, The Affiliated Hospital of Guizhou Medical University, Guiyang, Guizhou, China; 8 Department of Critical Care Medicine, Yijishan Hospital, The First Affiliated Hospital of Wannan Medical College, Wuhu, Anhui, China; 9 Department of Critical Care Medicine, The First Affiliated Hospital, China Medical University, Shenyang, Liaoning, China; 10 Key Laboratory of Chinese Internal Medicine of Ministry of Education and Beijing, Dongzhimen Hospital, Beijing University of Chinese Medicine, Beijing, China; 11 Dongzhimen Hospital, Beijing University of Chinese Medicine, Beijing, China; 12 Department of Critical Care Medicine, Tianjin Third Central Hospital, Tianjin, China; 13 Department of Critical Care Medicine, Northern Jiangsu People’s Hospital, Yangzhou, Jiangsu, China; 14 Department of Critical Care Medicine, Chinese PLA General Hospital, Beijing, China; 15 Department of Critical Care Medicine, The First Hospital of Jilin University, Changchun, Jilin, China; 16 Department of Emergency, China-Japan Friendship Hospital, Beijing, China; 17 Department of Critical Care Medicine, First People’s Hospital of Foshan, Foshan, Guangdong, China; 18 Department of Critical Care Medicine, The First Hospital of Lanzhou University, Lanzhou, Gansu, China; 19 Department of Emergency, Xinhua Hospital, School of Medicine, Shanghai Jiaotong University, Shanghai, China; 20 Department of Emergency, The First Affiliated Hospital of Henan Science and Technology University, Luoyang, Henan, China; 21 Hunan Provincial Institute of Emergency Medicine, Hunan Provincial People’s Hospital, Changsha, Hunan, China; 22 Department of Critical Care Medicine, The First Affiliated Hospital of Nanchang University, Nanchang, Jiangxi, China; 23 Department of Emergency, Shanghai General Hospital, School of Medicine, Shanghai Jiaotong University, Shanghai, China; 24 Department of Critical Care Medicine, Yantai Yuhuangding Hospital of Qingdao University, Yantai, Shandong, China; 25 Department of Critical Care Medicine, Beijing Friendship Hospital, Capital Medical University, Beijing, China; 26 Department of Critical Care Medicine, Qilu Hospital of Shandong University (Qingdao), Qingdao, Shandong, China; 27 Department of Critical Care Medicine, The First Affiliated Hospital of Zhengzhou University, Zhengzhou, Henan, China; 28 Department of Respiration, Chinese PLA General Hospital of Rocket Forces, Beijing, China; 29 Department of Anesthesiology, Xijing Hospital, Xi'an, Shaanxi, China; 30 Department of Critical Care Medicine, Jiangsu Province Hospital, Nanjing, Jiangsu, China; 31 Department of Critical Care Medicine, The Third Xiangya Hospital of Central South University, Changsha, Hunan, China; 32 Department of Critical Care Medicine, Yanbian University Hospital, Yanji, Jilin, China; 33 Department of Critical Care Medicine, Central Hospital of Shenyang Medical College, Shenyang, Liaoning, China; 34 Department of Critical Care Medicine, Yantaishan Hospital, Yantai, Shandong, China; 35 Department of Critical Care Medicine, The First Affiliated Hospital of Chongqing Medical University, Chongqing, China; 36 Department of Critical Care Medicine, Ruijin North Hospital, School of Medicine, Shanghai Jiaotong University, Shanghai, China; 37 Department of Critical Care Medicine, First Affiliated Hospital, Heilongjiang University of Chinese Medicine, Harbin, Heilongjiang, China; 38 Department of Emergency, First Affiliated Hospital of Kunming Medical University, Kunming, Yunnan, China; 39 Department of Emergency, Chinese PLA Wuhan General Hospital, Wuhan, Hubei, China; 40 Department of Critical Care Medicine, Shenzhen Traditional Chinese Medicine Hospital, Shenzhen, Guangdong, China; 41 Department of Emergency, Shanghai Sixth People’s Hospital, Shanghai, China; 42 Department of Critical Care Medicine, Zhongnan Hospital of Wuhan University, Wuhan, Hubei, China; 43 Department of Critical Care Medicine, Zhejiang Provincial People’s Hospital, Hangzhou, Zhejiang, China; 44 Department of Emergency, Zhongshan Hospital, Fudan University, Shanghai, China; 45 Department of Critical Care Medicine, The Affiliated Hospital of Qingdao University, Qingdao, Shandong, China; 46 Department of Emergency, Tianjin Medical University General Hospital, Tianjin, China; 47 Department of Critical Care Medicine, The Affiliated Hospital of Inner Mongolia Medical University, Hohhot, Inner Mongolia, China; 48 State Key Laboratory of Drug Research, Shanghai Institute of Materia Medica, Chinese Academy of Sciences, Zhangjiang Hi-Tech Park, Shanghai, China; 49 First Teaching Hospital of Tianjin University of Traditional Chinese Medicine, National Clinical Research Center for Chinese Medicine Acupuncture and Moxibustion, Tianjin, China; 50 Evidence-Based Medicine Center, Tianjin University of Traditional Chinese Medicine, Tianjin, China

## Abstract

**Importance:**

Previous research has suggested that Xuebijing injection (XBJ), an herbal-based intravenous preparation, may reduce mortality among patients with sepsis.

**Objective:**

To determine the effect of XBJ vs placebo on 28-day mortality among patients with sepsis.

**Design, Setting, and Participants:**

The Efficacy of Xuebijing Injection in Patients With Sepsis (EXIT-SEP) trial was a multicenter, randomized double-blind, placebo-controlled trial conducted in intensive care units at 45 sites and included 1817 randomized patients with sepsis (sepsis 3.0) present for less than 48 hours. Patients aged 18 to 75 years with a Sequential Organ Failure Assessment score of 2 to 13 were enrolled. The study was conducted from October 2017 to June 2019. The final date of follow-up was July 26, 2019. Data analysis was performed from January 2020 to August 2022.

**Interventions:**

The patients were randomized to receive either intravenous infusion of XBJ (100 mL, n = 911) or volume-matched saline placebo (n = 906) every 12 hours for 5 days.

**Main Outcomes and Measures:**

The primary outcome was 28-day mortality.

**Results:**

Among the 1817 patients who were randomized (mean [SD] age, 56.5 [13.5] years; 1199 [66.0%] men), 1760 (96.9%) completed the trial. In these patients, the 28-day mortality rate was significantly different between the placebo group and the XBJ group (230 of 882 patients [26.1%] vs 165 of 878 patients [18.8%], respectively; *P* < .001). The absolute risk difference was 7.3 (95% CI, 3.4-11.2) percentage points. The incidence of adverse events was 222 of 878 patients (25.3%) in the placebo group and 200 of 872 patients (22.9%) in the XBJ group.

**Conclusions and Relevance:**

In this randomized clinical trial among patients with sepsis, the administration of XBJ reduced 28-day mortality compared with placebo.

**Trial Registration:**

ClinicalTrials.gov Identifier: NCT03238742

## Key Points

**Question:**

Is Xuebijing injection (XBJ) effective in reducing mortality in patients with sepsis?

**Findings:**

In this randomized clinical trial that included 1817 patients with sepsis, the 28-day mortality rate was 18.8% in the XBJ group vs 26.1% in the placebo group, a significant difference.

**Meaning:**

Among patients with sepsis, treatment with XBJ, compared with the placebo group, resulted in lower 28-day mortality.

## Teaser

This randomized clinical trial assesses the effect of Xuebijing injection vs placebo on 28-day mortality among patients with sepsis.

## Introduction

Sepsis, a systemic disease that deteriorates rapidly with multiple organ dysfunction, is caused by dysregulated inflammatory response to infection and remains one of the leading causes of mortality worldwide.^1,2^ Numerous investigational therapies increasingly aimed at targeting the various biological pathways of sepsis have failed in large, randomized clinical trials, and none have shown a significant benefit on mortality.^3,4,5,6^

Xuebijing injection (XBJ), an herbal-based intravenous preparation, was licensed in 2004 by the National Medical Products Administration (NMPA, China) for the treatment of sepsis and multiple organ dysfunction syndrome.^7^ Xuebijing injection possesses an array of activities associated with its mechanism of protection in sepsis, and it can exert an antagonistic effect on endotoxin and an inhibitory effect on the uncontrolled release of endogenous inflammatory mediators produced by endotoxin-stimulated monocytes/macrophages.^8,9,10^ Furthermore, XBJ can improve the coagulation disorders present in disseminated intravascular coagulation,^11^ which is considered likely to be an important risk factor for mortality in sepsis. Previous studies have suggested that XBJ could also increase the activity of superoxide dismutase, regulate hypersensitive or hyposensitive immune responses, and prevent the development of organ dysfunction in acute insults.^12,13,14^

A well-conducted randomized study of patients with severe pneumonia showed that treatment with XBJ had impressive benefit.^15^ The study found that there was substantial difference in mortality between patients who received placebo and those who received XBJ. To our knowledge, this is the largest study yet to show there is benefit to using this herbal-based preparation. Two independent systematic reviews with meta-analyses (1144 patients and 3884 patients, respectively) also suggested, with a moderate certainty level of evidence, that XBJ was significantly associated with favorable outcomes and survival in patients with sepsis.^16,17^ However, there is still a lack of prospective outcome data from large confirmatory trials addressing this issue. Accordingly, this double-blind, placebo-controlled, multicenter study was conducted to determine the efficacy and the adverse effects of XBJ in addition to the standard care for patients with sepsis.

## Methods

### Study Design

The Efficacy of Xuebijing Injection in Patients With Sepsis (EXIT-SEP) trial was a randomized, double-blind, placebo-controlled, multicenter, parallel-group trial. The trial protocol (Supplement 1) has been previously published^18^ and is also available at ClinicalTrials.gov (NCT03238742). The trial followed the principles of the Declaration of Helsinki and was conducted in accordance with the Good Clinical Practices and Chinese regulations. This trial was approved by the Ethics Committee of Zhongda Hospital, Southeast University (2017ZDSYLL025-P01), and the institutional review board (or independent ethics committee) at each participating site. Written informed consent was obtained from all patients or legally authorized representatives. Any study-specific procedures were performed in accordance with all applicable ethical, regulatory, and local requirements. The trial was overseen by a blinded steering committee and an independent data and safety monitoring board. The steering committee oversaw the conduct and decision-making during the trial and made recommendations to the principal investigator. The data and safety monitoring board oversaw the safety of the trial. This study followed the Consolidated Standards of Reporting Trials (CONSORT) reporting guideline.

### Trial Sites and Patients

The trial was conducted in the intensive care units (ICUs) at 45 sites across China between October 2017 and June 2019, and final follow-up occurred in July 2019. All consecutive adult patients admitted in the ICUs and diagnosed with sepsis 3.0 were screened for enrollment. Patients aged 18 to 75 years were eligible for enrollment if they had a Sequential Organ Failure Assessment (SOFA) score of 2 to 13. Patients were excluded if they (1) were diagnosed with sepsis for more than 48 hours; (2) did not provide informed consent; (3) had severe primary disease, including unresectable tumors, hematologic diseases, and HIV infection; (4) had severe liver and kidney dysfunction (was defined as a liver or a renal component SOFA score ≥3 points); (5) receiving or had taken an immunosuppressant or had an organ transplant within the previous 6 months; (6) were pregnant or breastfeeding; and (7) had participated in other clinical trials in the previous 30 days.

### Randomization

For randomization, stratification was performed using predefined SOFA scores of 2 to 7 and 8 to 13 with a block size of 4 patients using an interactive voice/web response system, which linked the patients’ randomization numbers to treatment codes. Concealment of the randomized assignment was ensured by means of a centralized, secure, web-based system. These professionals had no interactions with the investigators, and the investigators were not aware of the block sizes and stratification.

### Intervention

The patients who met all the inclusion criteria and had none of the exclusion criteria were assigned randomly, in a 1:1 ratio, to receive 100 mL of XBJ (manufactured by Tianjin Chase Sun Pharmaceutical Co, Ltd, Z20040033) mixed with 100 mL of normal saline every 12 hours or matching placebo (200 mL) for 5 consecutive days. The chemical composition of XBJ is listed in the eAppendix in Supplement 2. These interventions were administered by a dedicated study nurse or a trained ICU nurse. The placebo and XBJ were supplied in identical labels. Opaque tubing and the covering of infusion bag with plastic sleeves were used to obscure any identifying features of the infusion. During the study, only the drug managers and study nurses who prepared the study drugs were aware of the allocation information of the patients, but these drugs managers and nurses did not participate in the data collection and outcome evaluation.

### Sepsis Management

The investigators followed the local sepsis management guidelines and the international guideline for management of sepsis and septic shock 2016.^19^ The early administration of antibiotics, maintenance of arterial blood pressure with a combination of volume resuscitation and vasopressors, and early treatment of the source of infection were recommended. Details of sepsis management were recorded in the case report form.

### Outcomes

The primary outcome was all-cause mortality 28 days after the randomization. The key secondary outcomes included were ICU and hospital mortality, ICU and hospital length of stay, 28-day ICU-free days (with a maximum of 28 days [best] and a minimum of 0 days [worst]), 28-day cumulative mechanical ventilation–free days, and change in Acute Physiology and Chronic Health Evaluation (APACHE) II score and the SOFA score change at day 3 and day 6. The SOFA scores in the trial ranged from 0 (normal organ function) to 24 (worst organ dysfunction). The change in the SOFA score was defined as a fixed-day SOFA score minus initial-day SOFA score. SOFA scores were calculated on days 3 and 6 in the EXIT-SEP trial. Detailed definitions of the outcomes are provided in the study protocol in Supplement 1.

The safety outcomes included any adverse events (AEs) and severe adverse events (SAEs) through 28 days of follow-up. The clinically significant laboratory abnormalities were required to be captured as AEs. These reports were analyzed by the study coordinating center together with the investigators.

### Data Collection and Monitoring

The investigators or staff entered the baseline characteristics, process variables, and outcome data into web-based case report forms for days 1 to 28 and reported any AEs to the trial’s sponsor. The patients, their surrogates, or their primary care physicians were contacted if additional data were needed. The trial data were monitored by independent monitors, and the data were monitored centrally by the staff from the coordinating center according to a prespecified monitoring plan in the study protocol in Supplement 1.

### Statistical Analysis

Based on the previous Chinese Epidemiological Study of Sepsis (CHESS) study^20^ conducted in China, a 28-day all-cause mortality rate of 24.3% in the placebo group was assumed in this trial. With a dropout rate of approximately 15%, a sample size of 1800 patients would provide a power of 80% for a 2-sided .05 significance level on an assumption of an absolute risk reduction of 6 percentage points in mortality.

The primary outcome was assessed by fitting a generalized linear model with a binomial distribution and identity link. The patients with an unknown mortality status at day 28 were not imputed for testing of the primary outcome. The same model was applied for other categorical variables, such as mortality (ICU, hospital). The Kaplan-Meier method and log-rank test were used to compare survival curves from randomization until 28 days. Prespecified subgroup analyses were performed for the following variables: age (<60 or ≥60 years), SOFA score (2-7 or 8-13), APACHE II score, mechanical ventilation, septic shock, continuous renal replacement therapy and ICU type. The results of these subgroup analyses are presented in a forest plot in eFigure 2 in Supplement 2.

Changes in the SOFA score and APACHE II score from baseline were analyzed by fitting linear mixed-effects models using the baseline value, treatment, visit, and treatment-by-visit interaction as fixed effects. The same approach without baseline adjustment was used for the other continuous variables, such as ICU-free days, mechanical ventilation–free days, and length of stay (ICU, hospital). The AE data were provided for descriptive purposes only.

To assess the robustness of the primary analyses, sensitivity analyses were performed. First, missing data for the primary outcome were imputed using the multiple imputation method under the missing-at-random assumption. Second, a prespecified analysis used a control-based pattern model to evaluate sensitivity to missing data departure from the assumption. A worse-case analysis and a tipping point analysis for the primary outcome were also performed.

All the efficacy analyses were performed in the intention-to-treat (ITT) population, which is defined as all randomized participants. Safety analyses were conducted for the patients in the ITT group who received at least 1 treatment session. Statistical analyses were performed using SAS, version 9.4 (SAS Institute) and R, version 3.4.1 (R Foundation), with a 2-sided *P* value less than .05 considered as significant. No adjustment was made for multiple comparisons; therefore, the secondary outcomes should be interpreted as exploratory.

## Results

### Study Population

**Figure 1.**
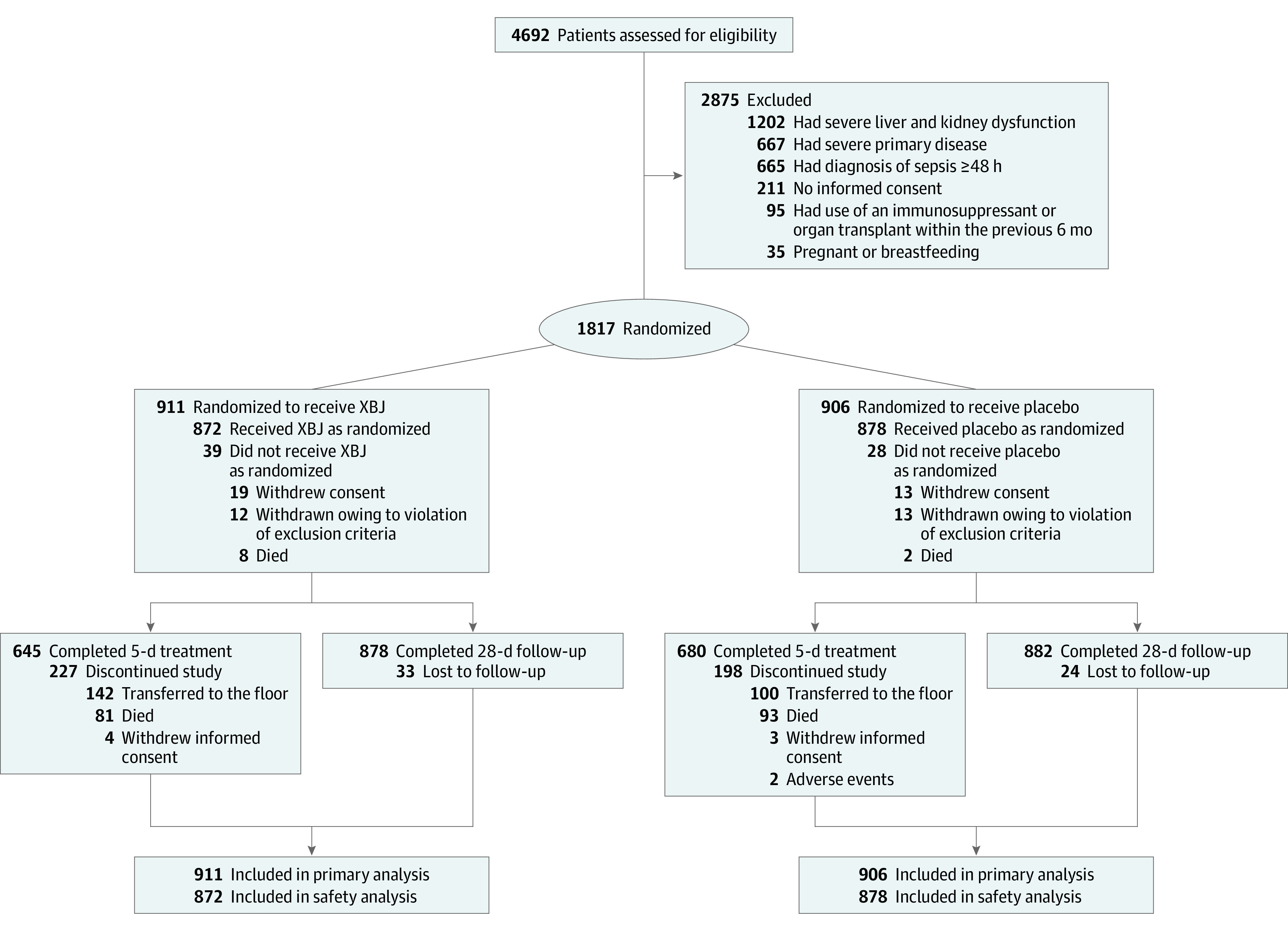
Flow of the Participants in the Efficacy of Xuebijing Injection for Sepsis (EXIT-SEP) Trial CONSORT flow diagram to show participant recruitment and follow-up. XBJ indicates Xuebijing injection.

Between October 20, 2017, and June 29, 2019, 4692 participants from 45 ICUs across 22 provinces in China were screened for eligibility, and 1817 (38.7%) were randomly allocated to receive either XBJ twice daily plus standard of care (n = 911) or placebo plus standard of care (n = 906) (Figure 1). The follow-up continued through July 26, 2019. Sixty-seven patients did not receive the assigned intervention because of death (n = 10), withdrawal of consent (n = 32), or withdrawal owing to violation of the exclusion criteria before receiving the intervention (n = 25). Of the remaining 1750 participants, 1325 (75.7%) completed the 5-day treatment period, and 425 (24.3%) discontinued the treatment during this study period. The reasons for discontinuation of the treatment are provided in Figure 1.

The baseline demographics, severity of illness, and comorbidities were well balanced between the study groups (Table 1). In both groups, the mean baseline SOFA score was approximately 7.1. The mean APACHE II score (range, 0-71; a higher score indicates greater severity of illness and higher risk of death) was approximately 12 in both groups. Almost half of the patients had septic shock (n = 830) at enrollment. The mean time from sepsis identification to randomization was 1.4 days. The primary sites of infection at baseline were comparable in the 2 groups (Table 1). The 2 most common sites of infection were the lung (814 of 1817, 44.8%) and intra-abdominal (584 of 1817, 32.1%). New infection after admission occurred in 134 (14.7%) patients in the XBJ group and 150 (16.6%) patients in the placebo group. As a concomitant medication, glucocorticoids were administered to approximately a quarter of the patients in both groups (eTable 1 in Supplement 2).

**Table 1.** Baseline Characteristics of Patients by Randomization Group

Characteristic	Placebo group (n = 906)	XBJ group (n = 911)
Age, mean (SD), y	56.8 (13.6)	56.3 (13.4)
Sex, No. (%)		
Men	619 (68.3)	580 (63.7)
Women	287 (31.7)	331 (36.3)
Han nationality^a^	887 (97.9)	888 (97.5)
BMI, mean (SD)	23.6 (2.9)	23.7 (3.1)
ICU types, No. (%)		
General	665 (73.4)	689 (75.6)
Emergency	202 (22.3)	192 (21.1)
Surgical	29 (3.2)	17 (1.9)
Respiratory	10 (1.1)	12 (1.4)
Primary site of infection, No. (%)		
Lung	396 (43.7)	418 (45.9)
Intra-abdominal	292 (32.2)	292 (32.1)
Urinary tract	53 (5.9)	60 (6.6)
Skin or soft tissue	31 (3.4)	26 (2.9)
Central nervous system	22 (2.4)	23 (2.5)
Blood	14 (1.6)	6 (0.7)
Other^b^	98 (10.8)	86 (9.4)
Source of infection, No. (%)		
Community-acquired	756 (83.4)	777 (85.3)
Nosocomial	150 (16.6)	134 (14.7)
Preexisting conditions, No. (%)		
Hypertension	270 (29.8)	267 (29.3)
Diabetes	193 (21.3)	166 (18.2)
Coronary artery disease	66 (7.3)	66 (7.2)
Stroke	54 (6.0)	52 (5.7)
Liver disease	38 (4.2)	46 (5.0)
Kidney disease	17 (1.9)	25 (2.7)
COPD	11 (1.2)	14 (1.5)
Malignant neoplasm	9 (1.0)	12 (1.3)
Other	127 (14.0)	127 (13.9)
SOFA score, mean (SD)^c^	7.1 (3.0)	7.1 (3.0)
Organ dysfunction, No. (%)^d^		
Respiratory	710 (78.4)	717 (78.7)
Coagulation	264 (29.1)	252 (27.7)
Hepatic	200 (22.1)	200 (22.0)
Cardiovascular	406 (44.8)	430 (47.2)
Neurologic	298 (32.9)	281 (30.9)
Kidney	150 (16.6)	163 (17.9)
APACHE II, mean (SD)^e^	12.7 (6.1)	12.5 (6.2)
<25	849 (96.7)	840 (96.3)
≥25	29 (3.3)	32 (3.7)
Heart rate, mean (SD), beats/min	101.4 (22.3)	102.0 (22.6)
Respiratory rate, mean (SD), breaths/min	21.6 (6.3)	21.9 (6.3)
Blood pressure, mean (SD), mm Hg		
Systolic	119.3 (23.9)	118.2 (23.8)
Diastolic	68.9 (14.4)	68.5 (15.1)
Medication within 48 h before randomization, No. (%)		
Glucocorticoid	129 (14.2)	121 (13.3)
Anticoagulant	228 (25.2)	212 (23.3)
Vasopressor	411 (45.4)	439 (48.2)
Antimicrobial agents	873 (96.4)	868 (95.3)
Antibacterial agents	866 (95.6)	866 (95.1)
Antifungal agents	74 (8.2)	66 (7.2)
Antivirals	70 (7.7)	53 (5.8)
Septic shock, No. (%)^f^	415 (47.3)	415 (47.6)
Mechanical ventilation, No. (%)^f^	492 (54.8)	483 (53.4)
CRRT, No. (%)^f^	93 (10.6)	79 (9.1)
Culture-proven pathogens, No. (%)^f^		
Gram positive	124 (14.7)	111 (13.0)
Gram negative	307 (36.5)	313 (36.5)
Gram positive and negative	49 (5.8)	40 (4.7)
Fungal	80 (9.5)	75 (8.8)
Viral	10 (1.2)	6 (0.7)
Atypical pathogens	1 (0.1)	2 (0.2)

Abbreviations: APACHE, Acute Physiology and Chronic Health Evaluation; BMI, body mass index, calculated as weight in kilograms divided by height in meters squared; COPD, chronic obstructive pulmonary disease; CRRT, continuous renal replacement therapy; ICU, intensive care unit; SOFA, Sequential Organ Failure Assessment; XBJ, Xuebijing injection.

^a^ As reported by the patient.

^b^ Other site of infection included unknown source.

^c^ The SOFA score includes subscores ranging from 0 to 4 for each of 6 components (respiratory, coagulation, liver, cardiovascular, neurologic, and renal components), with higher scores indicating more severe organ dysfunction.

^d^ Organ dysfunctions were defined as a SOFA score of 2 or higher for each of 6 components.

^e^ APACHE II scores range from 0 to 71; 0 indicates the lowest prediction of mortality and 71 indicates the highest. Data on APACHE II scores were available for 878 patients in the placebo group and 872 in the XBJ group.

^f^ Data on septic shock or CRRT were available for 878 patients in the placebo group and 872 in the XBJ group; data on culture-proven infection, for 841 and 857 patients, respectively; data on mechanical ventilation, for 898 and 904 patients, respectively.

### Primary Outcome

**Figure 2.**
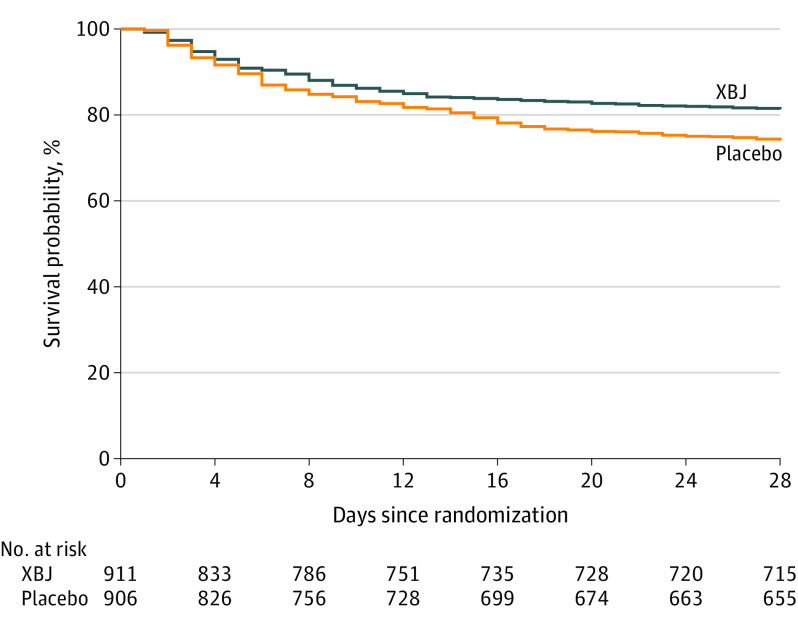
Probability of Survival From Randomization Through Day 28 Patients with unknown survival status at 28 days (n = 57) were censored on the last day they were known to be alive. XBJ indicates Xuebijing injection.

The 28-day mortality rate was 230 of 882 patients (26.1%) in the placebo group vs 165 of 878 patients (18.8%) in the XBJ group (*P* < .001), risk difference of 7.3 (95% CI, 3.4-11.2) percentage points. The Kaplan-Meier survival curve for the full analysis set is shown in Figure 2. Fifty-seven patients (33 in the XBJ group and 24 in the placebo group) had an unknown mortality status at day 28 and were assessed as assumed alive by the investigators. Similar results were confirmed in the 2 sensitivity analyses (eTable 2 in Supplement 2), worse-case analysis (eTable 3 in Supplement 2), and tipping point analysis (eFigure 1 in Supplement 2).

### Secondary Outcomes

There was a significant between-group difference in the ICU mortality after randomization (placebo, 20.0%, vs XBJ, 14.2%; risk difference, 5.8 [95% CI, 2.2-9.2] percentage points; *P* = .001) or in hospital mortality after randomization (placebo, 22.8%, vs XBJ, 17.0%; risk difference, 5.8 [95% CI, 2.1-9.5] percentage points; *P* = .002), or in ICU-free days, or in 28-day cumulative mechanical ventilation–free days (Table 2). The results of the secondary outcomes were considered exploratory only.

**Table 2.** Primary and Secondary Outcomes^a^

Variable	Placebo group (n = 906)	XBJ group (n = 911)	Difference (95% CI)	*P* value
**Primary outcome** ^b^				
28-d Mortality, No./total No. (%)	230/882 (26.1)	165/878 (18.8)	7.3 (3.4 to 11.2)	<.001
**Secondary outcomes** ^c^				
Mortality, No./total No. (%)				
ICU	176/882 (20.0)	125/878 (14.2)	5.8 (2.2 to 9.2)	.001
Hospital	201/882 (22.8)	149/878 (17.0)	5.8 (2.1 to 9.5)	.002
Length of stay, mean (95% CI), d^d^				
In ICU	10.6 (10.1 to 11.0)	9.8 (9.3 to 10.3)	0.8 (0.1 to 1.5)	.03
In hospital^e^	15.4 (14.8 to 16.0)	15.9 (15.3 to 16.5)	0.5 (−0.3 to 1.4)	.23
28-d Cumulative mechanical ventilation–free days, mean (95% CI), d^f^	17.7 (16.9 to 18.4)	19.5 (18.8 to 20.2)	−1.8 (−2.9 to −0.8)	<.001
28-d ICU-free days, mean (95% CI), d^g^	12.4 (11.7 to 13.0)	14.4 (13.8 to 15.1)	−2.1 (−3.0 to −1.2)	<.001
Change, SOFA score, mean (95% CI)^h^				
3-d	−0.9 (−1.1 to −0.7)	−1.0 (−1.2 to −0.8)	0.1 (−0.2 to 0.4)	.55
6-d	−1.7 (−1.9 to −1.5)	−2.4 (−2.6 to −2.1)	0.7 (0.4 to 1.0)	<.001
Change, APACHE II score, mean (95% CI)^h^				
3-d	−2.0 (−2.3 to −1.7)	−2.1 (−2.4 to −1.8)	0.1 (−0.4 to 0.5)	.75
6-d	−2.3 (−2.6 to −1.9)	−2.8 (−3.2 to −2.4)	0.5 (0.001 to 1.0)	.046

Abbreviations: APACHE, Acute Physiology and Chronic Health Evaluation; ICU, intensive care unit; SOFA, Sequential Organ Failure Assessment; XBJ, Xuebijing injection.

^a^ For rows including No./total No., the total number refers to the number of patients with valid data.

^b^ A total of 57 patients were not collected due to lost to follow-up.

^c^ Missing data not imputed for secondary outcomes analyses.

^d^ Data were calculated using a mixed-effects model.

^e^ The length of stay in the hospital included the length of stay in the ICU.

^f^ The mechanical ventilation–free days were defined as the total number of days a patient was alive and not on mechanical ventilation from randomization to 28 days. Data were calculated using a generalized linear model.

^g^ The ICU-free days were defined as the number of days alive and free of ICU from randomization to 28 days. Data were calculated using a generalized linear model.

^h^ Negative changes indicate better outcomes. Data were calculated using a repeated-measures mixed-effects model–adjusted baseline values.

### Subgroup Analysis

We performed efficacy analyses across subgroups by age, baseline SOFA score, baseline APACHE II scores, mechanical ventilation, septic shock, ICU types, and continuous renal replacement therapy. In subgroup analyses, the difference in the 28-day mortality rates between the placebo and XBJ groups was 7.6 (95% CI, 3.2-12.0) percentage points in the subgroup of patients with baseline SOFA score of 2 to 7 (n = 972) and 6.8 (95% CI, 0.3-13.3) percentage points in the subgroup of patients with SOFA score of 8 to 13 (n = 788) (eFigure 2 in Supplement 2). The findings were broadly consistent between the ITT population and subgroup analyses.

### AE Analyses

Overall, 422 patients (24.1%) experienced at least 1 AE (200 patients [22.9%] in the XBJ group and 222 patients [25.3%] in the placebo group) within 28 days of follow-up. The non–end point AEs are enumerated in eTable 4 in Supplement 2. The most common AE in the XBJ group was elevated alanine aminotransferase level (51 of 872, 5.8%). Other AEs in the XBJ group included elevated aspartate aminotransferase level (38 of 872, 4.4%) and high white blood cell count (36 of 872, 4.1%). There were similar distributions of AEs in the XBJ and placebo groups with no reports of drug-related SAEs. There were no treatment discontinuations due to drug toxicity.

## Discussion

This large-scale randomized clinical trial found that patients with sepsis treated with XBJ had significantly lower mortality at 28 days compared with placebo. The main findings from the subgroup analyses were consistent with the primary analysis. In addition, XBJ could also reduce ICU mortality and was associated with cumulative mechanical ventilation–free days and ICU-free days in patients with sepsis during the 28-day follow-up period, as compared with placebo. While this study was powered to detect differences in the secondary outcomes, the statistically significant difference in these secondary outcomes should be cautiously interpreted considering that there were 10 secondary outcomes without adjustment for multiple comparisons. There were no statistically significant differences in the occurrence of AEs between the 2 groups.

Progress in the development of novel therapeutics to treat sepsis has come to a virtual standstill.^21^ While enormous strides have been made in the understanding of basic molecular mechanisms underlying the pathophysiology of sepsis, a distressingly long list of novel therapeutic agents has been tested in clinical trials over the past 30 years without a single, specific agent showing consistent morality benefit in sepsis trials.^22,23^ The study may have identified a clinically important benefit made by XBJ—a 7.3-percentage-point reduction in 28-day mortality with statistical significance. The trial was prospectively powered to detect a 6-percentage-point difference in 28-day survival based on the results of the CHESS analysis.^20^ The investigators may consider a 5-percentage-point difference in 28-day mortality to be clinically significant, and this study succeeded in detecting this difference. Notably, a clear mortality difference between other countries and China was again shown in the EXIT-SEP trial. The mortality finding of the placebo group is strongly consistent with that of the nationwide CHESS study.^20^ The 28-day placebo mortality was 17.0% in the ADDRESS trial,^24^ 30.8% in the PROWESS trial,^25^ 31.6% in the LOVIT trial,^26^ and 29.4% in the SCARLET trial,^27^ indicating that the EXIT-SEP patients were indeed moderately to severely ill.

There are several possible reasons for this finding. Sepsis trials are particularly challenging due to the heterogeneity of the patient population. Based on the previous analyses, the entry criteria written into the current clinical trial protocol was a SOFA score of 2 to 13. The mortality rate was 74.3% in patients with a SOFA score of more than 13 in the Chinese population.^20^ Thus, mortality may be substantially influenced by factors that are not captured by a SOFA score greater than 13. Considering the above reasons, the present study further quantified the consistency of SOFA change to reflect the treatment effects on mortality. Additionally, the experience of vasopressin and septic shock trials suggested that the sepsis 3.0 definition we adopted might decrease the sample size by half and increase the 28-day mortality rates by approximately 10%.^28^ Finally, the appropriate sample size contributed to obtaining the accurate, statistically significant results. The mortality rate in the placebo group (26.1%) was similar to the assumed value used for the sample size calculation prior to the study (24.3%).

Recent studies have indicated that there are anti-inflammatory, anticoagulation, and immunoregulatory effects of moderate XBJ in animal models of sepsis.^29,30^ Clinical evidence was provided by the limited studies that were focused on the improvement of organ dysfunction,^31,32^ but there has been a lack of conclusive effect on mortality thus far. In patients with sepsis, independent meta-analysis found significant association with XBJ use; however, the small sample sizes and limited methodological quality may have affected the robustness and credibility of these findings.^16,17^ Studies on the mechanisms underlying XBJ’s protective effects and previous data analyses jointly summarized the 3 crucial success factors: (1) the early use of XBJ, (2) an optimal duration of 5 days, (3) and in an appropriate population. All of these factors were successfully implemented in the current trial.

One of the main results of our study was the similar rate of AEs in the XBJ group compared with the placebo group. No additional AE occurrence was identified in this study. Furthermore, no serious AEs were observed to be associated with XBJ, which was consistent with those of the previous reports.^31,33^ At least one reason may be responsible for the low rate of XBJ-associated SAEs in the patients with sepsis in the current trial. First, 93.0% of the patients with APACHE II scores less than 25 had a reduced risk. The exclusion of patients who had high risk could further account for the lack of SAEs in our trial. It is noteworthy that other AEs were rare in both of the groups in our study. Every year, 250 000 patients are treated with XBJ in China. The results from a postauthorization safety study indicate that XBJ is safe and well tolerated, and most of the adverse drug reactions were relatively mild or nonserious.^34^

### Limitations

This study has several limitations. First, patient loss to follow-up from the trial, which occurred at a slightly higher rate in the XBJ group (3.6%), during the trial may have affected the estimates of the treatment effect. Second, the study was performed in a single country, which limited the generalizability of our results. Third, the patients were essentially those with sepsis not severe enough to qualify for a high risk of death. This estimation of high risk of death was generally defined as an APACHE II score of 25 or more. This trial was underpowered to detect the differences in mortality among specific subgroups of APACHE II score of 25 or greater. As such, any subgroup analysis should be interpreted as exploratory. Fourth, whether the enrolled patient population represented a typical sepsis patient population with regard to its clinical heterogeneity needs to be further explored. The greater proportion of patients with respiratory infection than intra-abdominal infection in this study was not entirely consistent with previous observations. Additionally, the proportion of male patients was higher than that of female patients in the placebo group. Fifth, the prespecified analyses that were performed to investigate the population that was most likely to respond favorably to XBJ treatment were preplanned, and this still does not eliminate selective publication of results that fit a hypothesis. Sixth, antibiotic therapy was administrated before randomization, and the effectiveness of antimicrobial drugs may potentially affect the anti-inflammatory effect of XBJ. Seventh, the long-term survival of the patients administered XBJ was not evaluated (the final follow-up has been completed, but the data collection is still ongoing; therefore, the long-term mortality has not yet been analyzed). Additional trials of XBJ for patients with sepsis using long-term mortality as a primary outcome could further inform whether XBJ carries an enhanced therapeutic effect for this population.

## Conclusions

In this randomized clinical trial of patients with sepsis, an intravenous infusion of XBJ at a dose of 100 mL every 12 hours for 5 days significantly reduced 28-day mortality and had a safety profile that was acceptable within the context of this clinical trial.

## Notes

Supplement 1.
Trial Protocol

Supplement 2.
eAppendix
**eFigure 1.** Tipping point analysis for the Primary Outcome **eFigure 2.** Subgroup Analysis of the Primary Outcome **eTable 1.** Use of Other Medications for Sepsis During Study **eTable 2.** Sensitivity Analyses for the Primary Outcome **eTable 3.** Worse-case Analysis for the Primary Outcome **eTable 4.** Overall Summary of Adverse Events in Safety Population

Supplement 3. **Nonauthor Collaborators.** EXIT-SEP Investigators

Supplement 4.
Data Sharing Statement
