# Supplementary material for: Effect of an Herbal-Based Injection on 28-Day Mortality in Patients With Sepsis: The EXIT-SEP Randomized Clinical Trial
Source: JAMA Intern Med. 2023 May 1;183(7):647–55. doi: 10.1001/jamainternmed.2023.0780 (PMC10152378; doi:10.1001/jamainternmed.2023.0780)
Supplement: Supplement 1. — Trial Protocol [file jamainternmed-e230780-s001.pdf]

CLINICAL STUDY PROTOCOL for EXIT-SEP

Efficacy of Xuebijing Injection in Patients with Sepsis: A Multicentre, Randomized, Blind, Placebo-controlled Trial (EXIT-SEP TRIAL)

Study Code: EXIT-SEP

Principal investigator: Haibo Qiu, Zhongda Hospital, Southeast University

SUMMARY OF THE TRIAL

**Title**	**Efficacy of Xuebijing Injection in Patient with Sepsis: A Multicentre, Randomized, Blind, Placebo-controlled Trial (EXIT-SEP)**
**Study design**	**A multi-center, randomized, placebo-controlled, double-blind clinical trial of Xuebijing Injection in patients with sepsis.**
**Primary Objective**	**To evaluate whether Xuebijing Injection, when administered to subjects with sepsis, can reduce 28-day all-cause mortality.**
**Study Center(s)**	**Up to 45 study centers**
**Number of Subjects (planned)**	**Approximately 1800 randomized subjects**
**Subjects**	**<u>Inclusion criteria</u>**<br>1. **Subjects is diagnosed with sepsis 3.0**<br>2. **Age ≥18 and 75**<br>3. **Subjects had a Sequential Organ Failure Assessment (SOFA) score of 2-13**<br>4. **Subjects must be receiving treatment in an intensive care unit (ICU)**<br>5. **Informed consent obtained from patient or acceptable patient surrogate**<br><br>**<u>Exclusion criteria</u>**<br>1. **Subjects is diagnosed with sepsis for more than 48 hours**<br>2. **Female who is pregnant or lactating at time of admission**<br>3. **Had severe primary disease, including unresectable tumours, haematological diseases and human immunodeficiency virus (HIV) infection, had severe liver and kidney dysfunction (was defined as liver or kidney component SOFA score ≥ 3 points)**<br>4. **Receiving/used an immunosuppressant, an organ transplant within the previous 6 months**<br>5. **Participated in other clinical trials in the previous 30 days**
**Treatments**	**Treatment group: Xuebijing injection (100 ml, every 12 hours) diluted in 100 ml 0.9% sodium chloride injection**<br>**Placebo group: 0.9% sodium chloride injection (200 ml, every 12 hours)**
**Duration of Treatment**	**Active treatment will continue for 5 days, discharge from study hospital, discharge from the ICU, study withdrawal, or death, whichever comes first.**
**Follow-up**	**All participants will be followed for a total of 28 days.**
**Informed Consent**	**Explicit written, signed informed consent from the subject or legally authorized representative will be obtained prior to any protocol specific procedures**
**Randomization**	**Subjects will be randomly assigned in a 1:1 fashion to receive Xuebijing Injection or placebo. Randomization occurs immediately after baseline SOFA score via a real-time, internet-based randomization method.**
**Primary outcome**	**28-day all-cause mortality**
**Secondary outcome**	**1) In ICU mortality**<br>**2) In hospital mortality**<br>**3) Length of stay (LOS) in the ICU**

	**4) LOS in hospital, 28-day cumulative mechanical ventilation-free days**<br>**5) 28-day ICU-free days**<br>**6) The change in SOFA score on day 3**<br>**7) The change in SOFA score on day 6**<br>**8) The change in Acute Physiology and Chronic Health Evaluation (APACHE) II score on day 3**<br>**9) The change in APACHE II score on day 6**
**Safety outcome**	**Incidence of serious adverse events (SAEs)**<br>**Incidence of adverse events (AEs)**
**Study Timeline**	**Recruitment period estimated in 36 months**<br>**Follow-up per patient: 28 days**

PROTOCOL SIGNATURE PAGE PRINCIPAL INVESTIGATOR

STUDY CODE: EXIT-SEP

Study titled: Efficacy of Xuebijing Injection in Patient with Sepsis: A Multicentre, Randomized, Blind, Placebo-controlled Trial (EXIT-SEP)

The signatures on this page indicate review and approval of the final version of the protocol.

By signing this document we confirm that the clinical study will be conducted in accordance with the protocol and all applicable laws and regulations including, but not limited to, the International Conference on Harmonization Guideline for Good Clinical Practice (GCP) and the ethical principles that have their origins in the Declaration of Helsinki.

Signature: ___________ Haibo Qiu **May 10, 2017**

Professor Haibo Qiu **Date of signature**

Principal investigator

Table of Contents

ABBREVIATIONS 7

1. INTRODUCTION 8

2. TRIAL OBJECTIVES 12

3. OVERALL STUDY DESIGN 13

3.1 Study design 13
3.2 Study population 13
3.3 Inclusion criteria 13
3.4 Exclusion criteria 13

4. STUDY PROCEDURES 13

4.1 Summary of study treatment 13
4.2 Study drug 14
4.3 Randomization 14
4.4 Blinding 15
4.5 Duration of Study Treatment 15
4.6 Concomitant Treatment 16
4.7 Interventions prohibited during the trial 17

5. OUTCOMES 17

5.1 Primary outcome 17
5.2 Secondary outcomes 17
5.3 Safety outcomes 18

6. ETHICS AND DISSEMINATION 18

6.1 Guiding Principles 18
6.2 Ethical Considerations 18
6.3 Informed Consent Form 19
6.4 Liability Insurance for Drug Clinical Trials 19
6.5 Privacy Protection of Subjects 19
6.6 Dissemination Policy 19

7. DATA MANAGEMENT 20

7.1 Data Collection Methods 20

7.2 Data Collection Methods 20

7.3 Data Quality and Monitoring 21

7.4 Protocol Deviations 21

8. STATISTICAL CONSIDERATIONS 22

8.1 Power Calculation and Sample Size 22

8.2 Analysis of Results 22

9. SAFETY MONITORING AND REPORTING 22

9.1 Data Monitoring Committee 22

9.2 Adverse event (AE) / Adverse reaction (AR) 23

9.3 Serious adverse event (SAE) / Serious adverse reaction (SAR) 23

9.4 Reporting AEs/ARs and SAEs/SARs 24

9.5 Contact phone numbers for SAE advice 24

9.6 Post-Trial Care 24

10. FUNDING 24

References 25

ABBREVIATIONS

AE Adverse event

AR Adverse reaction

APACHE Acute physiology and chronic health evaluation

CRF Case report form

DIC Disseminated intravascular coagulation

eCRF Electronic case report form

GCP Good clinical practice

HREC Human research ethics committee

ICU Intensive care unit

MODS Multiple organ dysfunction syndrome

RCT Randomized controlled trial

SAE Serious adverse event

SAR Serious adverse reaction

SOD Superoxide dismutase

SOFA Sequential organ failure assessment

Efficacy of Xuebijing Injection in Patients with Sepsis: A Multicentre, Randomized, Blind, Placebo-controlled Trial

EXIT-SEP PROTOCOL

1. INTRODUCTION

Despite significant advances over the past several decades, mortality from sepsis remains unacceptably high. A study suggested global estimates of 31.5 million sepsis and 19.4 million severe sepsis cases, with potentially 5.3 million deaths annually [1]. A retrospective cohort study of adult patients revealed sepsis in 6% of adult hospitalizations, of which 15.0% died in the hospital [2]. Even though the mortality of septic shock has declined over the past 10 years along with the advancement of the medical industry, the overall mortality of septic shock still reaches up to 40% [3].

The introduction of new Sepsis-3 criteria [4] has facilitated clinician differentiation of sepsis from common infections and prescription of effective treatment and management in a timely manner. However, the available therapeutic strategy for sepsis is limited to antibiotic administration and supportive care, since current therapeutic measures targeting septic pathological processes (such as inflammatory response, blood coagulation and immunologic dysfunction) are less promising. Therefore, exploring effective therapeutic medications for decreasing the severity and improving the prognosis of sepsis is of significant scientific and social value.

Sepsis is involved in a complex neuroimmune-endocrine response to pathogens instead of pathogen-induced direct damage to local tissues. In addition to inflammatory and anti-inflammatory responses, infection can also lead to abnormalities in endocrine function, metabolism, blood coagulation, and eventually organ dysfunction. Among all pathological changes, the inflammatory response and immune dysfunction play a critical role in the development of sepsis [5]. The state of immunoactivation is not sustained during the process of sepsis. Indeed, a study has shown that the initial hyperinflammatory response in

sepsis (manifested by excessive secretion of inflammatory mediators) is quickly followed by the development of a sustained anti-inflammatory or immunosuppressive state. Moreover, such proinflammatory and immunosuppressive responses might also coexistin the early stage of sepsis.

In the initial stage, infection activates phagocytic cells and facilitatestheir enhanced phagocytic and killing functions. These activated phagocytic cells secrete excessive cytokines (e.g., IL-1, IL-6, IL-8, IL-12, and TNF-α), which subsequently induce an inflammatory response and blood vessel dilatation and eventually increase blood vessel permeability. Moreover, activated macrophages can act as professional antigens and induce specific immune responses. Along with disease progression, the proliferative ability of lymphocytes gradually declines, which manifests as immune responses dominated by T helper lymphocytes (Th2) and excessive lymphocyte apoptosis. When massive apoptosis of immune cells (including T cells, B cells, macrophages, and dendritic cells) is induced by excessive inflammatory factors[6], immune effector cells can only secrete fewer cytokines, become hyporesponsive and present impaired antigen presenting capability. As a consequence of these immunosuppressive processes, the body loses “immunological surveillance”, becomes less sensitive to pathogens with immune dysfunction, and eventually develops multiple organ dysfunction syndrome (MODS) or even death.

Previously, immunotherapy against inflammatory mediators has failed to improve survival in patients with sepsis. Different types of anti-inflammatory medications, such as interleukin-1 receptor antagonist, anti-bradykinin, anti-TNF antibody, soluble TNF receptor, platelet activating factor receptor antagonist, platelet activating factor, acetylhydrolase and anti-prostaglandin, were tested in preclinical and clinical trials. However, none of these agents could significantly improve patient survival in clinical trials, even with acceptable results in preclinical trials[7]. It seems that simple anti-inflammatory mediators cannot reverse the pathophysiological changes related to sepsis since patients also have immune dysfunction.

The status of immune function determines the prognosis of patients with sepsis. With the constant in-depth exploration of septic pathogenesis, immunoregulation therapies have

gained increasing attention. Recent clinical studies have shown that the regulation of immune function may improve the prognosis of patients with sepsis.

The immune function status of sepsis might vary among patients with different basal states or under different disease courses. Immune enhancement, the apparent inflammatory response and tissue damage are predominant in the early stage of sepsis[8]. Moreover, when HLA-DR expression and inflammatory factor production by monocytes cannot recover for sepsis patients ina severe stage, immune paralysis status significantly worsens the prognosis [9]. A study showed that an immunoregulator, thymosin alpha 1 (Zadaxin), can improve clinical outcomes for patients with elderly severe sepsis [10]. Comparatively, for young sepsis patients, the anti-inflammatory agent ulinastatin might also improve the prognosis and shorten hospitalization duration and ICU stay [11].

Therefore, either an excessive inflammatory response or unbalanced immune function prevails, and abnormal host responses to infection are considered the most fundamental cause of organ failure in patients with sepsis. Hence, regulation of the host response in patients is currently an important target for exploring new drugs and therapies. Xuebijing injection could serve as an effective therapeutic agent for sepsis due to its effects of inhibiting inflammation, improving immunity and regulating the coagulation balance.

Xuebijing injection was prepared with Carthami Flos (Hong Hua, 红花), Paeoniae Radix Rubra (Chi Shao, 赤芍), Chuanxiong Rhizoma (Chuan Xiong, 川芎), Salviae Miltiorrhizae Radix Et Rhizoma (Dan Shen, 丹参) and Angelicae Sinensis Radix (Dang Gui, 当归). Pharmacological studies have demonstrated that Xuebijing injection might inhibit endotoxin, control the uncontrolled release of endogenous inflammatory mediators produced by endotoxin-induced monocytes/macrophages, improve the coagulation function in disseminated intravascular coagulation (DIC), increase superoxide dismutase activity, regulate both hyper and hypoimmunoresponses, and protect and repair damaged organs under stress. In summary, Xuebijing injection can antagonize endotoxin; inhibit inflammatory mediators; improve immune function; restore coagulation balance; and protect tissues and organs.

A study has shown that Xuebijing injection protects major animal organs after severe injury mainly by inhibiting the synthesis and release of high mobility group box 1 (HMGB-1), a late-acting inflammatory mediator, and subsequently preventing the occurrence and progression of sepsis and MODS or improving the prognosis[12].

Xuebijing can effectively release the proliferation-inhibitory status of septic effector T cells (Teff) by inducing the apoptosis of regulatory T cells (Treg) through the Caspase3/9 pathway and then significantly regulating the immune disorder caused by sepsis. It can also promote the proliferative activity of T cells through CD11clowCD45RBhighDC, improve cellular immune function, and ultimately reduce mortality. In addition, Xuebijing can promote M2 polarization of macrophages, improve the inflammatory response and prognosis, and reduce mouse mortality[13].

Xuebijing injection can also improve coagulation and endothelial function. Animal experiments have demonstrated that Xuebijing injection significantly reduces the levels of plasma IL-6 and TNF-α in rats with sepsis by reducing the tissue factors (TFs) of monocytes and downregulating protease-activated receptors (PAR-1), improves coagulation function, and recovers platelet function [14]. In an experiment using qualitative detection with Tachypiens amebocyte lysate (TAL), Xuebijing injection exhibited a direct inactivation effect on endotoxin in vitro. More specifically, the minimum antitoxic concentrations for the 1-hour and 4-hour reactions were 0.125 mg/ml and 0.063 mg/ml, respectively. Xuebijing injection reduces endotoxin-induced lethality in mice.

In DIC model rats, it improves coagulation function and increases the levels of platelets and fibrinogen. It also shortens the thrombin time (TT) and prothrombin time (PT) and increases the platelet aggregation rate. Moreover, it reduces plasma thromboxane B2 (TX B2) levels. Additionally, it has a therapeutic effect on endotoxin-induced toxic injury of the rat liver and can increase superoxide dismutase (SOD) activity. Furthermore, it has an antagonistic effect on the increase in tumour necrosis factor-α (TNF-α) serum levels in endotoxin-stimulated mice, and it can increase serum anti-goat red blood cell antibody levels to improve humoural immune function in sensitized mice accordingly. Additionally, it increases the clearance index K, the phagocytic index α, and the phagocytic activity of the reticuloendothelial system in normal mice [15].

Clinical studies have also shown that Xuebijing injection inhibits the activation of the inflammatory response during sepsis, reduces the occurrence of coagulation dysfunction, alleviates the development of sepsis and improves the prognosis of patients with sepsis [16]. A systematic review of Xuebijing injection for the treatment of sepsis recruited 25 articles, including 1970 cases (1022 cases in the Xuebijing group and 948 cases in the control group), and considered 28-day mortality as the main evaluation indicator. The results showed that the 28-day mortality for patients receiving both Xuebijing injection and conventional treatment [relative risk (RR)=0.65, 95% CI 0.54 - 0.79] was lower than that for patients receiving only conventional treatment, and the improvement in APACHE II scores after 7 days and 14 days of treatment was better than that in the conventional treatment group (WMD = 3.74, 95% CI 2.05 - 5.43; WMD = 10.50, 95% CI 1.43 - 19.57, respectively). Therefore, this systematic review demonstrated that Xuebijing injection could reduce the 28-day mortality of patients with sepsis and improve the APACHE II score [17]. Another randomized controlled trial also showed that Xuebijing injection could markedly reduce the severity of patients with sepsis, promote organ function recovery, regulate coagulation dysfunction and improve the prognosis in a safe manner [18].

Anti-sepsis medication still relies on antibiotics, and no medicine can be provided for definite improvement of the mortality of sepsis patients. Xuebijing injection may improve the prognosis of sepsis patients by regulating immune inflammation and coagulation. Sepsis-3 diagnostic criteria focus more on the impact of organ dysfunction on the prognosis of patients, while Xuebijing injection may increase the survival rate of patients with sepsis by improving organ function. Therefore, it is necessary to conduct a multicentre, blind, randomized and controlled clinical study to evaluate the effect of Xuebijing injection for sepsis patients with 28-day all-cause mortality as the primary endpoint.

2. TRIAL OBJECTIVES

To evaluate whether Xuebijing Injection, when administered to subjects with sepsis, can reduce 28-day all-cause mortality.

3. OVERALL STUDY DESIGN

3.1 Study design

This is a prospective, multi-centre, randomized, blind, placebo controlled trial.

3.2 Study population

Patients admitted to any ICU of the participating hospitals with the primary diagnosis of sepsis 3.0 will be screened for inclusion into this study.

3.3 Inclusion criteria

1) Subjects is diagnosed with sepsis 3.0
2) A ge ≥18 years and ≤75 years
3) Subjects had a Sequential Organ Failure Assessment (SOFA) score of 2-13
4) Subjects must be receiving treatment in an intensive care unit (ICU)
5) Informed consent obtained from patient or legally authorized representatives

3.4 Exclusion criteria

1) Subjects is diagnosed with sepsis for more than 48 hours
2) Female who is pregnant or lactating at time of admission
3) Had severe primary disease, including unresectable tumors, hematological diseases and human immunodeficiency virus (HIV) infection, had severe liver and kidney dysfunction (was defined as a liver or a kidney component SOFA score ≥ 3 points)
4) Receiving/took an immunosuppressant, an organ transplant within the previous 6 months
5) Participated in other clinical trials in the previous 30 days

4. STUDY PROCEDURES

4.1 Summary of study treatment

All eligible ICU patients will be enrolled as soon as possible after fulfilling the criteria for randomization. Patients will be allocated in a 1:1 ratio to either the treatment group, receiving Xuebijing Injection, or to control group, receiving placebo.

4.2 Study drug

1) Xuebijing Injection

Tianjin Chase Sun Pharmaceutical Co., Ltd. (National Medicine Permit No. Z20040033)

Xuebijing injection is prepared by Carthami Flos (*Hong Hua*, 红花), Paeoniae Radix Rubra (*Chi Shao*, 赤芍), Chuanxiong Rhizoma (*Chuan Xiong*, 川芎), Salviae Miltiorrhizae Radix Et Rhizoma (*Dan Shen*, 丹参) and Angelicae Sinensis Radix (*Dang Gui*,当归). Glucose and polysorbate 80 are also added as excipients (for injection).

This product is a clear pale brown liquid.

[Specification] Ten millilitres of Xuebijing injection per ampoule; 5 ampoules per pack.

[Storage] Sealed and stored in a cool (≤20°C), dry place away from light.

[Package] Xuebijing injection is packed with a low borosilicate glass ampoule. The secondary package is the "Xuebijing injection package for scientific purposes".

2) Placebo

0.9% sodium chloride injection, consisting of 250 ml in-service products packaged in subsites; HANACO® lightproof infusion set and light shield, provided by the manufacturer for free.

4.3 Randomization

This study adopts a third-party interactive web response system (IWRS), which is implemented as competitive enrolment with a randomized block design. The random process is blinded to the investigators. Subjects are assigned to the treatment group and the placebo group at a 1:1 ratio.

When the screened subjects are eligible for recruitment, investigators at subsites log in to the IWRS and enter the subjects' basic information, including their screening numbers and initials, according to the system prompts. The IWRS randomly assigns the subjects SOFA scores (2-7 points vs. 8-13 points) as a stratification factor. The SSID (6 digits) and random number (4 digits) of each subject are unique and bound to each other.

After random numbers are generated, drug managers log in to the IWRS again for the allocation results and then provide this information to study nurses and dispense the investigational products to study nurses for instillation.

4.4 Blinding

Investigators and drug managers have independent access to the IWRS for login and operation. The blinded code is concealed by the IWRS to prevent investigators from obtaining it. The case report form (CRF) only indicates the random number of a subject without group information. Investigators and evaluators do not know the subject group information during the study and evaluation. The investigational drugs are infused withopaque brown bags and tubing to prevent investigators and other participants in the study from knowing the group information.

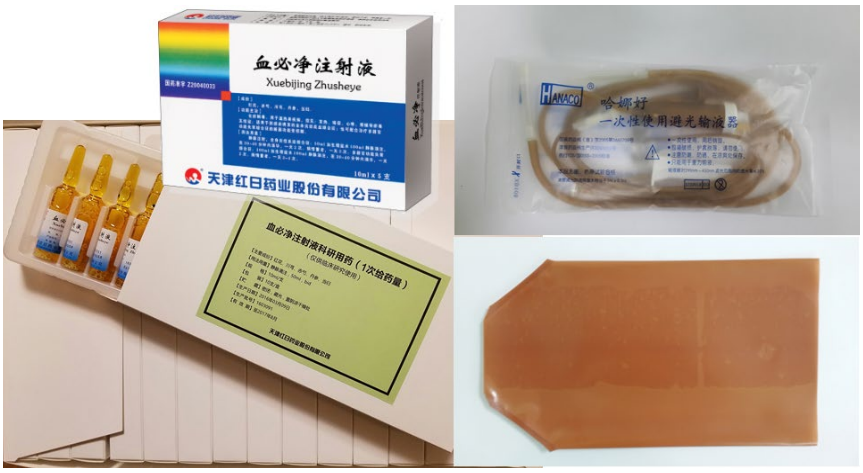

Figure 1. Blinding of the drug used in the Xuebijing group and placebo group

The trial drug covered by opaque brown bags and tubing (Figure 1). During the study, only drug managers and study nurses who prepared the study drugs are aware of the allocation information of subjects, but they do not participate in the data collection and outcome evaluation of the study. All study participants should sign a letter of confidentiality commitment to avoid disclosing blinded treatment information.

4.5 Duration of Study Treatment

Treatment will continue until one of the following criteria for treatment cessation is met:

1) For Xuebijing group-5 days of Xuebijing Injection treatment has been administered
2) For control group -5 days of placebo has been administered

3) Patient is discharged from ICU
4) Death occurs
5) Serious adverse events suspected to be related to a study medication develops
6) Development of a contraindication to any of the study drugs
7) Consent has been withdrawn or consent to continue has not been granted

4.6 Concomitant Treatment

All patients enrolled in this study will be managed by a standardized approach which will include the following elements:

International Guidelines for Management of Sepsis and Septic Shock: 2016, including early fluid resuscitation, anti-infection, hormone usage, anticoagulation therapy, intravenous nutrition, and other therapeutic methods. The use of anti-infective agents, hormones, and anticoagulants should be recorded in detail: the generic names, dosage, number of doses per day, and duration.

Anti-infective agents: Antiviral agents and antifungal medications can be prescribed in accordance with the methods recommended by relevant guidelines and expert consensus; antibacterial agents should be prescribed in accordance with the recommended methods of Surviving Sepsis Campaign: International Guidelines for Management of Sepsis and Septic Shock: 2016;

Circulation support: Blood pressure should be maintained above 90 mmHg/60 mmHg, and mean arterial pressure should be ≥ 65 mmHg; Vasopressors could be prescribed depending on the conditions of the disease, including dopamine, dobutamine, epinephrine, norepinephrine, etc., and norepinephrine should be prioritized. The dose of vasopressors should be recorded at any time upon usage.

Nutritional support: Enteral nutrition should be prioritized for patients with mechanical ventilation, and intravenous nutrition can be prescribed when the following indications occur: Patients cannot ingest food through the gastrointestinal tract due to oesophageal fistula, intestinal fistula, gastrointestinal malformation, or other conditions; Serious trauma or inflammation of the gastrointestinal tract; Serious gastrointestinal dysfunction, such as ulcerative colitis, gastrointestinal haemorrhage, and other conditions;

Ingestion through the gastrointestinal tract is dangerous, such as tracheoesophageal fistula, laryngeal insufficiency, and other conditions. These patients should receive deep intravenous feeding.

The ICU team will have full and independent control of patient management, which will not be affected by participation in the study.

4.7 Interventions prohibited during the trial

1) During the treatment period, ulinastatin are prohibited.
2) Ulinastatin and Xuebijing injection are prohibited from the end of the treatment period to the 28-day follow-up.

5. OUTCOMES

5.1 Primary outcome

28-day all-cause mortality

Twenty-eight (28) day mortality is the most commonly used measure by scientific and regulatory authorities to assess efficacy of treatment of sepsis, and that will be the primary outcome of the study.

5.2 Secondary outcomes

**Secondary outcomes**	
**ICU mortality,**	The proportion of patients who had died to ICU discharge
**Hospital mortality**	The proportion of patients who had died to hospital discharge
**Length of stay (LOS) in the ICU**	The total duration of stay in the ICU for the first 28 days after randomization
**LOS in hospital**	The total duration of stay in the hospital for the first 28 days after randomization
**28-day cumulative mechanical ventilation-free days**	The total number of days a patient was alive and not on mechanical ventilation support from randomization to 28 days
**28-day ICU-free days**	The number of days alive and free of ICU from randomization to 28 days
**The change in SOFA score on day 3**	The change in SOFA score from baseline score measured at randomization to the score at Day 3
**The change in SOFA score on day 6**	The change in SOFA score from baseline score measured at randomization to the score at Day 6

**The change in Acute Physiology and Chronic Health Evaluation (APACHE) II score on day 3**	The change in APACHE II score from baseline score measured at randomization to the score at Day 3
**The change in APACHE II score on day 6**	The change in APACHE II score from baseline score measured at randomization to the score at Day 6

5.3 Safety outcomes

1) Incidence of serious adverse events (SAEs)
2) Incidence of adverse events (AEs)

6. ETHICS AND DISSEMINATION

6.1 Guiding Principles

This study is to be performed in accordance with the ethical principles of the Declaration of Helsinki (June 1964 and amended 1975, 1983, 1989, 1996, 2000, 2008, 2013 and Note of Clarification 2002 and 2004), China GCP Notes for Guidance on Good Clinical Practice (GCP).

6.2 Ethical Considerations

Ethical review procedure: All subsites engaged in this study must be reviewed and approved by the ethics committee of the hospital before study initiation. The amendment of the study protocol and informed consent form should be submitted to the ethics committee for re-review and re-approval;

Ethics committee review of the sponsor's organization: Prior to study initiation, the principal investigator should submit the investigator's brochure, study protocol, informed consent form, CRF, and qualifications and resumes of the investigators, and other major study materials to the ethics committee of the study sponsor's organization for review and approval;

Ethics committee review of the subsite organization: The principal investigator of each subsite should submit the approval letter issued by ethics committee of the sponsor's organization, the investigator's brochure, study protocol, informed consent form, CRF, qualifications and resumes of the principal investigator from the subsite, and other study data to the ethics committee of the hospital for review and approval;

When reviewing and approving study documents, the ethics committee must confirm the study title and indicate the version number, version date and review date of the reviewed study documents.

6.3 Informed Consent Form

The investigator must inform the subject or his/her legal representative of the information about the study in both oral and written ways. The subjects or their legal representatives have the right to know the detailed information about this study.

The informed consent form (ICF) must be reviewed and approved by the ethics committee. If necessary, the investigator is responsible for explaining the contents of the ICF to the subject in a manner that the subject can understand. The subject or his/her legal representative should have sufficient time to read and understand the ICF before signing it.

The ICF must be signed and dated by the subject or his/her legal representative. The signed ICF should be kept separately by the investigator and the subject, and the investigator should keep the original copy on file for monitoring and audit.

6.4 Liability Insurance for Drug Clinical Trials

To provide better protection for the rights and interests of subjects, all subjects will be insured with "Ping An Clinical Trial Liability Insurance" before study initiation. A copy of the insurance certificate will be submitted to the ethics committee of each subsite for filing.

6.5 Privacy Protection of Subjects

The investigator takes the responsibility to maintain the anonymity of subjects and keep documents showing the identity of subjects strictly confidential. In CRFs or other study documents, subjects can only be identified with codes. The investigator must properly maintain a screening form that records the subject code, name, and home address.

6.6 Dissemination Policy

A writing committee will be formed to review and publish the data from the study. This committee will consist of the Steering Committee and a subset of investigators. The writing committee will write/review all drafts of abstracts and full-length manuscripts and will choose the appropriate journal (for manuscripts) or meeting (for abstracts) for submission.

The EXIT-SEP Steering Committee commits that when the study is completed, the data from this study will be published, regardless of the outcome of the study.
All information concerning the EXIT-SEP trial supplied to the investigators by the Steering Committee and not previously published is considered confidential and shall remain the sole property of the EXIT-SEP Steering Committee. The investigator agrees to use this information only in accomplishing the study and will not use it or the data generated from the study for other purposes without first obtaining written authorization from the EXIT-SEP Steering Committee.

7. DATA MANAGEMENT

7.1 Data Collection Methods

All data will be collected by trained staff at each study site using a case report form (CRF) worksheet developed by the coordinating centre. Data will then be entered into the web database (electronic case report form [eCRF]). Randomized patients will be followed up to death or 28 days post-randomization whichever occurs first. Study day 0 commences on randomization and concludes at the expiry of the calendar day. Data collection will be restricted primarily to those variables necessary to define clinical patient characteristics including: baseline demographics, primary diagnoses, physiological parameters, diagnostic interventions, therapeutic interventions and documentation of deaths and other SAEs.

7.2 Data Collection Methods

CRF design: The data management organization will draft the CRF and submit it to the principal investigator for review and approval.
Database design: The data management organization will be responsible for designing the electronic database and developing the procedure for data logic verification per the study protocol. The data management organization shall test the database with simulated data or real CRF data.
Study data input: The study data input is the responsibility of the investigator. To ensure the accuracy of the data, the investigator needs to fill in the paper copy of the CRF carefully and enter its content into the electronic CRF. Problems found in the

entry process should be recorded and reported to the CRA and data management organization in a timely manner to take measures promptly.

Data verification

If an error occurs during the data entry process, the logic verification programme will mark the erroneous data entered in red, prompting the person inputting the data to make corresponding modifications.

The CRA is responsible for verifying the consistency of the paper CRF and electronic CRF, monitoring the quality of entries and analysing and handling existing problems.

Database lock: The principal investigator of all subsites will sign on the completed data and confirm it. After CRA verification, they will sign and confirm it. Once all the signed documents meet the requirements of the study protocol and the blinded review meeting is held, the data management organization will lock the database (no modification can be performed after database lock) and export clean data after the database is locked. Problems found after the data are locked will be modified in the statistical analysis programme after confirmation. All errors and modifications should be recorded and properly maintained.

7.3 Data Quality and Monitoring

Several procedures to ensure data quality and protocol standardization will help to minimize bias and to optimize data quality. The study will be monitored by quality control reviews of protocol compliance, data queries, safety reporting and protocol deviations. On-site monitoring will only be performed on a case by case basis if quality control issues are flagged by electronic review of data Medical records, any other relevant source documents and the site investigator files must be made available to the monitoring representative for these monitoring visits during the study and at the completion of the study as needed.

7.4 Protocol Deviations

A protocol deviation is an unanticipated or unintentional departure from the expected conduct of an approved study that is not consistent with the current research protocol or consent document. A protocol deviation may be an omission, addition or change in any procedure described in the protocol. Given that the investigator is responsible for patient

safety and care he/she may implement a deviation from, or a change of the protocol to eliminate an immediate hazard to trial patients without prior Human Research Ethics Committee approval. The implemented deviation or change must be reported in a protocol deviation form. The deviation must be reported via the study website by the principal investigator. A summary of protocol deviations will be reported to the coordinating Human Research Ethics Committee; a serious breach will be reported ASAP.

8. STATISTICAL CONSIDERATIONS

8.1 Power Calculation and Sample Size

Based on the previous observational CHESS study conducted in China, a 28-day all-cause mortality rate of 24.3% in the placebo group was assumed in this trial. With a dropout rate of approximately 15%, a sample size of 1800 patients provided 80% power at the two-sided 0.05 significance level on an assumption of an absolute risk reduction of 6%in mortality.

8.2 Analysis of Results

A senior statistician at Dongzhimen Hospital will perform data analysis on an intention-to-treat basis. Summary statistics will be used to describe the clinical data and presented as mean ± SD, or percentages as appropriate. Continuous variables will be compared using a two-sample t-test or Wilcoxon rank-sum test if data show serious deviations from a normal distribution. Categorical data or ordinal data will be compared using a Wilcoxon rank-sum test, chi-square test or Fisher's exact test, as appropriate. All tests will be two-sided. Statistical significance declared for probability values of 0.05 or less. Analysis of the outcome of excluded patients due to other trials etc. will be in accordance with the CONSORT guidelines.

9. SAFETY MONITORING AND REPORTING

9.1 Data Monitoring Committee

An independent Data Monitoring Committee (DMC), consisting of experts in intensive care, clinical research and biostatistics will be established before patient enrolment and will review all trial protocols. The role of the DMC will be to provide study oversight to ensure

that the rights and safety of patients involved in the study are protected by reviewing reported AEs and making recommendations to the Management Committee (MC). Intensive care patients experience a number of common aberrations in laboratory values, signs and symptoms due to the severity of the underlying disease and the impact of standard therapies. Intensive care patients will frequently develop life-threatening organ failure(s) unrelated to study interventions and despite optimal management. Therefore, consistent with established practice in academic ICU trials, events that are part of the natural history of the primary disease process or expected complications of critical illness will not be reported as SAEs in this study. All AEs which are considered to be potentially causally related to the study intervention or are otherwise of concern in the investigator's judgment will be reported.

9.2 Adverse event (AE) / Adverse Reaction (AR)

An AE is any untoward medical occurrence in a clinical trial participant administered a medicinal product and does not necessarily have a causal relationship with this treatment. It is recognized that the patient population with critical illness will experience a number of common aberrations in laboratory values, signs and symptoms due to the severity of the underlying illness and the impact of standard therapies. These will not necessarily constitute an AE unless they are considered to be of concern or related to the study or the intervention in the investigator's clinical judgment. In all cases, the condition or disease underlying the symptom, sign or laboratory value should be reported. An adverse reaction is defined as any untoward and unintended response to an investigational medicinal product related to any dose administered. All AEs judged by either the reporting investigator or the sponsor as having a reasonable possibility of a causal relationship to an investigational medicinal product will qualify as adverse reactions. All of the investigational drugs for this trial are approved drugs in China.

9.3 Serious adverse events (SAE) / Serious adverse reactions (SAR)

A SAE or serious adverse reaction is defined as any adverse event/reaction that: Results in death; Is life-threatening; Requires hospitalization or prolongation of current hospitalization; Results in persistent or significant disability or incapacity; Death is an

expected outcome among patients with septic shock, therefore, death will not be considered a SAE. Standard care of patients with septic shock includes a host of complications that fit the definition of an SAE. Medical and scientific judgment will be exercised by the site principal investigator in deciding whether an adverse event/ reaction will be classified as serious in other situations to avoid over reporting.

Specific serious adverse reaction: Suspected or confirmed anaphylactic or anaphylactoid reaction towards one of the study drugs

9.4 Reporting AEs/ARs and SAEs/SARs

Adverse events/reactions and serious adverse events/reactions will be recorded on a separate case report form. SAEs should be reported to the coordinating centre within 24 hours of study staff becoming aware of the event. The coordinating centre staff will be responsible for following-up all events to ensure all details are available.

The coordinating centre is also responsible for reporting directly to HRECs and investigators, who forward any relevant information to their institution. Copies of any reporting and correspondence to and from the local HREC should also be sent to the coordinating centre.

9.5 Contact phone numbers for SAE advice

Chief Investigator (Prof Haibo Qiu): +86-25-83262551

9.6 Post-Trial Care

If post-trial care for compensation to those who suffer harm from trial participation will be covered by Ping An Clinical Trial Liability Insurance.

10. FUNDING

This study is funded by the Development Center for Medical Science and Technology, National Health Commission of the People's Republic of China (WK-2016-HR-03).

None of the funding organizations will contribute to the study design; collection, management, analysis and interpretation of data; writing of the report or the decision to submit the report for publication.

References

[1] Fleischmann C, Scherag A, Adhikari NK, et al. Assessment of Global Incidence and Mortality of Hospital-treated Sepsis. Current Estimates and Limitations. Am J Respir Crit Care Med. 2016;193(3):259-72.

[2] Sameer S Kadri, Chanu Rhee, Jeffrey R Strich, et al. Estimating Ten-Year Trends in Septic Shock Incidence and Mortality in United States Academic Medical Centers Using Clinical Data. Chest. 2017;151(2):278-285.

[3] Chanu Rhee, Raymund Dantes, Lauren Epstein, et al. Incidence and Trends of Sepsis in US Hospitals Using Clinical vs Claims Data, 2009-2014. JAMA. 2017;318(13):1241-1249.

[4] Singer M, Deutschman CS, Seymour CW, et al. The Third International Consensus Definitions for Sepsis and Septic Shock (Sepsis-3). JAMA. 2016;315(8):801–810.

[5] Kaukonen KM, Bailey M, Pilcher D, Cooper DJ, Bellomo R. Systemic inflammatory response syndrome criteria in defining severe sepsis. N Engl J Med. 2015. 372(17): 1629-38.

[6] Hotchkiss RS, Nicholson DW. Apoptosis and caspases regulate death and inflammation in sepsis. Nat Rev Immunol. 2006. 6(11): 813-22.

[7] Eichacker PQ, Parent C, Kalil A, et al. Risk and the efficacy of antiinflammatory agents: retrospective and confirmatory studies of sepsis. Am J Respir Crit Care Med. 2002. 166(9): 1197-205.

[8] Hotchkiss RS, Opal S. Immunotherapy for sepsis--a new approach against an ancient foe. N Engl J Med. 2010. 363(1): 87-9.

[9] Henry G Gomez, Sandra M Gonzalez, Jessica M Londoño, et al. Immunological characterization of compensatory anti-inflammatory response syndrome in patients with severe sepsis: a longitudinal study. Crit Care Med. 2014;42(4):771-80.

[10] Wu J, Zhou L, Liu J, et al. The efficacy of thymosin alpha 1 for severe sepsis (ETASS): a multicenter, single-blind, randomized and controlled trial. Crit Care. 2013. 17(1): R8.

[11] Karnad DR, Bhadade R, Verma PK, et al. Intravenous administration of ulinastatin (human urinary trypsin inhibitor) in severe sepsis: a multicenter randomized controlled study. Intensive Care Med. 2014. 40(6): 830-8.

[12] Chen S, Dai G, Hu J, et al. Discovery of Xuebijing Injection Exhibiting Protective Efficacy on Sepsis by Inhibiting the Expression of HMGB1 in Septic Rat Model Designed by Cecal Ligation and Puncture. 2016;23(6):e1819-e1825.

[13] Liu YC, Yao FH, Chai YF, et al. Xuebijing Injection Promotes M2 Polarization of Macrophages and Improves Survival Rate in Septic Mice. Evid Based Complement Alternat Med. 2015. 2015: 352642.

[14] Gao YL, Chai YF, Yao YM. Advancement in the research of mechanism of immune dysfunction in sepsis and the regulatory effects of Xuebijing injection. Zhonghua Shao Shang Za Zhi. 2013;29(2):162-5. Chinese.

[15] Xu Q, Liu J, Guo X, et al. Xuebijing injection reduces organ injuries and improves survival by attenuating inflammatory responses and endothelial injury in heatstroke mice. BMC Complement Altern Med. 2015;15:4.

[16] Qin Yin, Chunsheng Li. Treatment effects of xuebijing injection in severe septic patients with disseminated intravascular coagulation. Evid Based Complement Alternat Med. 2014;2014:949254.

[17] Hu Jing, Shang Hongcai, Li Jing, et al. A Systematic Evaluation of Xuebijing Injection in the Treatment of Patients with Sepsis. Medical Journal of Chinese People's Liberation Army. 2010. (1): 9-12.

[18] Chen Yunxia,Li Chunsheng. A Randomized Controlled Multi-center Study of Xuebijing Injection in the Treatment of Patients with Sepsis. Chinese Journal of Emergency Medicine. 2013. (2): 130-135.

Protocol Amendments

SUBJECT: Summary of Changes to Protocol 1.1: *Efficacy of Xuebijing Injection in Patients with Sepsis: A Multicentre, Randomized, Blind, Placebo-controlled Trial (EXIT-SEP TRIAL)*

This is a summary of Protocol Changes (1 amendment) during the study. The changes were made under the applicable section of the protocol was updated accordingly.

Change in Section(s) & Page Number(s)	Initial wording in Protocol Version 1.0	Amended or New wording in Protocol Version 1.1
Outcomes section, page 3[#]	Secondary outcomes will be the improvement of SOFA scores, the improvement of the Acute Physiology and Chronic Health Evaluation II score (the improvement means the difference of score between the day enrolled in the study and the 6 days), duration of mechanical ventilation, mortality in the ICU and duration of stay in the ICU.	Secondary outcome<br>1) In ICU mortality<br>2) In hospital mortality<br>3) Length of stay (LOS) in the ICU<br>4) LOS in hospital, 28-day cumulative mechanical ventilation-free days<br>5) 28-day ICU-free days<br>6) The change in SOFA score on day 3<br>7) The change in SOFA score on day 6<br>8) The change in Acute Physiology and Chronic Health Evaluation (APACHE) II score on day 3<br>9) The change in APACHE II score on day 6

[#]Liu S, Yao C, Zhang J, Yang Y, Qiu H; EXIT-SEP Investigators. Efficacy of Xuebijing Injection for Sepsis (EXIT-SEP): protocol for a randomised controlled trial. BMJ Open. 2019 Aug 28;9(8):e028664.
