# Supplementary material for: Effect of an Herbal-Based Injection on 28-Day Mortality in Patients With Sepsis: The EXIT-SEP Randomized Clinical Trial
Source: JAMA Intern Med. 2023 May 1;183(7):647–55. doi: 10.1001/jamainternmed.2023.0780 (PMC10152378; doi:10.1001/jamainternmed.2023.0780)
Supplement: Supplement 2. — eAppendix eFigure 1. Tipping point analysis for the Primary Outcome eFigure 2. Subgroup Analysis of the Primary Outcome eTable 1. Use of Other Medications for Sepsis During Study eTable 2. Sensitivity Analyses for the Primary Outcome eTable 3. Worse-case Analysis for the Primary Outcome eTable 4. Overall Summary of Adverse Events in Safety Population [file jamainternmed-e230780-s002.pdf]

Supplemental Online Content

Liu S, Yao C, Xie J, et al; for the EXIT-SEP Investigators. Effect of an herbal-based injection on 28-day mortality in patients with sepsis: the EXIT-SEP randomized clinical trial. *JAMA Intern Med.* Published online May 1, 2023. doi:10.1001/jamainternmed.2023.0780

eAppendix

eFigure 1. Tipping Point Analysis for the Primary Outcome

eFigure 2. Subgroup Analysis of the Primary Outcome

eTable 1. Use of Other Medications for Sepsis During Study

eTable 2. Sensitivity Analyses for the Primary Outcome

eTable 3. Worse-Case Analysis for the Primary Outcome

eTable 4. Overall Summary of Adverse Events in Safety Population

This supplemental material has been provided by the authors to give readers additional information about their work.

© 2023 Liu et al. *JAMA Internal Medicine.*

eAppendix

The chemical composition of Xuebijing injection

ID	Compound	Molecular Mass	Molecular Formula	Average content level
		Da		*µmol/L*
Constituents originating from the component herbs Chuanxiong/Danggui only				
**1**	Senkyunolide I	224.1049	$C_{12}H_{16}O_4$	293.1±28.1
**2**	Senkyunolide H	224.1049	$C_{12}H_{16}O_4$	64.8±6.3
**3**	Senkyunolide G	208.1099	$C_{12}H_{16}O_3$	44.5±3.1
**4**	Senkyunolide N	226.1205	$C_{12}H_{18}O_4$	40.8±5.2
**5**	3-Hydroxy-3-*n*-butylphthalide	206.0943	$C_{12}H_{14}O_3$	37.6±4.8
**6**	*Z*-6,7-Epoxyligustilide	206.0943	$C_{12}H_{14}O_3$	12.7±0.5
**7**	6,7-Dihydroxyligustilide	224.1049	$C_{12}H_{16}O_4$	10.9±1.1
**8**	Senkyunolide A	192.1150	$C_{12}H_{16}O_2$	4.8±2.6
**9**	Senkyunolide J	226.1205	$C_{12}H_{18}O_4$	3.4±0.4
**10**	4-Hydroxy-3-*n*-butylphthalide	206.0943	$C_{12}H_{14}O_3$	2.4±0.3
Constituents originating from the component herb Chishao only				
**11**	Mudanpioside F	344.1471	$C_{16}H_{24}O_8$	5.8±1.0
**12**	1-*O*-β-D-Glucopyranosyl-Paeonisuffrone	360.1420.	$C_{16}H_{24}O_9$	1.6±0.5
**13**	Desbenzoylpaeoniflorin	376.1369	$C_{16}H_{24}O_{10}$	22.1±13.7
**14**	Albiflorin	480.1632	$C_{23}H_{28}O_{11}$	102.4±27.6
**15**	Paeoniflorin	480.1632	$C_{23}H_{28}O_{11}$	2470±142.8
**16**	Oxypaeoniflorin	496.1581	$C_{23}H_{28}O_{12}$	112.9±7.6
**17**	Oxypaeoniflorin isomer	496.1581	$C_{23}H_{28}O_{12}$	4.0±0.8
**18**	Ortho-oxypaeoniflorin	496.1581	$C_{23}H_{28}O_{12}$	4.3±0.7
**19**	Mudanpioside E	526.1686	$C_{24}H_{30}O_{13}$	11.3±1.1
**20**	6'-*O*-Galloyl-desbenzoylpaeoniflorin	528.1479	$C_{23}H_{28}O_{14}$	1.3±0.5
**21**	Benzoylpaeoniflorin	584.1894	$C_{30}H_{32}O_{12}$	58.3±6.8
**22**	Benzoyloxypaeoniflorin	600.1843	$C_{30}H_{32}O_{13}$	1.9±0.2
**23**	Mudanpioside C	600.1843	$C_{30}H_{32}O_{13}$	1.6±0.4
**24**	Mudanpioside J	630.1949	$C_{31}H_{34}O_{14}$	2.0±0.3
**25**	Galloylpaeoniflorin	632.1741	$C_{30}H_{32}O_{15}$	56.0±13.7
**26**	Isomer of galloylpaeoniflorin or galloylalbiflorin	632.1741	$C_{30}H_{32}O_{15}$	7.2±1.5

© 2023 Liu et al. *JAMA Internal Medicine.*

**27**	Isomer of galloylpaeoniflorin or galloylalbiflorin	632.1741	$C_{30}H_{32}O_{15}$	2.0±0.6
**28**	Galloyloxypaeoniflorin	648.1690	$C_{30}H_{32}O_{16}$	2.4±0.4
Constituents originating from the component herb Danshen only				
**29**	Protocatechuic aldehyde	138.0	$C_7H_6O_3$	139.3±28.7
**30**	Protocatechuic acid	154.0	$C_7H_6O_4$	14.4±1.8
**31**	Tanshinol	198.1	$C_9H_{10}O_5$	66.4±15.5
**32**	Salvianolic acid G	340.1	$C_{18}H_{12}O_7$	0.1±0.0
**33**	Rosmarinic acid	360.1	$C_{18}H_{16}O_8$	40.4±6.3
**34**	Salvianic acid C	378.1	$C_{18}H_{18}O_9$	0.3±0.0
**35**	Salvianic acid C isomer	378.1	$C_{18}H_{18}O_9$	0.3±0.0
**36**	Salvianolic acid D	418.1	$C_{20}H_{18}O_{10}$	0.8±0.3
**37**	Isosalvianolic acid C	492.1	$C_{26}H_{20}O_{10}$	36.0±6.2
**38**	Salvianolic acid C	492.1	$C_{26}H_{20}O_{10}$	3.7±0.9
**39**	Salvianolic acid A	494.1	$C_{26}H_{22}O_{10}$	2.7±0.4
**40**	Salviaflaside	522.1	$C_{24}H_{26}O_{13}$	0.8±0.1
**41**	Lithospermic acid	538.1	$C_{27}H_{22}O_{12}$	1.2±0.2
**42**	Salvianolic acid J	538.1	$C_{27}H_{22}O_{12}$	1.9±0.3
**43**	Salvianolic acid B	718.1	$C_{36}H_{30}O_{16}$	23.9±3.1
**44**	Salvianolic acid E	718.1	$C_{36}H_{30}O_{16}$	1.3±0.3
**45**	4-Methoxylsalvianolic acid B	732.2	$C_{37}H_{32}O_{16}$	0.1±0.0
Constituents originating from the component herb Honghua only				
**46**	Saffloquinoside D	612.169	$C_{27}H_{32}O_{16}$	36.7±3.2
**47**	Saffloquinoside C	612.169	$C_{27}H_{32}O_{16}$	30.0±2.7
**48**	Hydroxysafflor yellow A	612.169	$C_{27}H_{32}O_{16}$	751.2±39.5
**49**	Safflomin C	614.1636	$C_{30}H_{30}O_{14}$	11.3±1.5
**50**	Saffloquinoside E	614.1636	$C_{30}H_{30}O_{14}$	6.7±1.1
**51**	Carthamin	910.2168	$C_{43}H_{42}O_{22}$	0.1±0.0
**52**	Anhydrosafflor yellow B	1044.2747	$C_{48}H_{52}O_{26}$	3.6±1.2
**53**	Kaempferol	286.0477	$C_{15}H_{10}O_6$	1.0±0.2
**54**	Kaempferol 3-glucoside	448.1006	$C_{21}H_{20}O_{11}$	9.0±1.3
**55**	Kaempferol 3-rha-(1-6)-glucoside	594.1585	$C_{27}H_{30}O_{15}$	45.6±15.4
**56**	Kaempferol 3-glc-(1-2)-glucoside	610.1534	$C_{27}H_{30}O_{16}$	14.9±2.5
**57**	3-Glc-kaempferol 7-glucuronide or 6-Glc-6-hydroxyapigenin 7-glucuronide	624.1326	$C_{27}H_{28}O_{17}$	12.2±5.6
**58**	6-Hydroxykaempferol 7-glucoside	464.0955	$C_{21}H_{20}O_{12}$	11.2±6.5

© 2023 Liu et al. *JAMA Internal Medicine.*

**59**	6-Hydroxykaempferol 3-glucoside	464.0955	$C_{21}H_{20}O_{12}$	0.3±0.1
**60**	6-Hydroxykaempferol 3-rha-(1-6)-glucoside	610.1534	$C_{27}H_{30}O_{16}$	3.6±2.0
**61**	6-Hydroxykaempferol 3,6-diglucoside	626.1483	$C_{27}H_{30}O_{17}$	2.2±1.1
**62**	6-Hydroxykaempferol 6,7-diglucoside	626.1483	$C_{27}H_{30}O_{17}$	0.9±0.1
**63**	6-Glc-6-Hydroxykaempferol 3-rha-(1-6)-glucoside	772.2062	$C_{33}H_{40}O_{21}$	9.0±4.2
**64**	6-Hydroxykaempferol 7-(3,6-diglc)-glucoside	788.2011	$C_{33}H_{40}O_{22}$	7.0±2.2
**65**	6-Hydroxykaempferol 7-(3,6-diglc)-glucuronide	802.1804	$C_{33}H_{38}O_{23}$	1.5±0.6
**66**	Quercetin	302.0427	$C_{15}H_{10}O_7$	0.7±0.1
**67**	Quercetin 3-glucoside	464.0955	$C_{21}H_{20}O_{12}$	5.6±1.3
**68**	Quercetin 3-rha-(1-6)-glucoside	610.1534	$C_{27}H_{30}O_{16}$	10.0±3.3
**69**	3-Rha-quercetin 7-glucuronide	624.1326	$C_{27}H_{28}O_{17}$	0.7±0.3
**70**	Quercetin 3,7-diglucoside	626.1483	$C_{27}H_{30}O_{17}$	22.5±7.5
**71**	Eriodictyol	288.0634	$C_{15}H_{12}O_6$	0.8±0.1
**72**	Neocarthamin	450.1162	$C_{21}H_{22}O_{11}$	10.6±2.1
**73**	Neocarthamin isomer	450.1162	$C_{21}H_{22}O_{11}$	13.8±2.2
**74**	5,6,7,4'-Tetrahydroxyflavone 6,7-diglucoside	612.169	$C_{27}H_{32}O_{16}$	47.3±11.5
**75**	Apigenin	270.0528	$C_{15}H_{10}O_5$	0.4±0.1
**76**	6-Hydroxyapigenin	286.0477	$C_{15}H_{10}O_6$	0.7±0.2
**77**	Luteolin	286.0477	$C_{15}H_{10}O_6$	0.3±0.0
**78**	Luteolin 7-glucoside	448.1006	$C_{21}H_{20}O_{11}$	0.9±0.2
**79**	Scutellarin	462.0798	$C_{21}H_{18}O_{12}$	2.5±1.5
**80**	Safflochalconeside	432.1056	$C_{21}H_{20}O_{10}$	0.1±0.0
Constituents originating from multiple component herbs of XueBiJing				
**81**	p-Coumaric acid	164.0473	$C_9H_8O_3$	69.0±23.1
**82**	4-Glucosyloxybenzoic acid	300.0845	$C_{13}H_{16}O_8$	20.9±2.3
**83**	p-Hydroxybenzoic acid	138.0317	$C_7H_6O_3$	52.1±7.0
**84**	Chlorogenic acid	354.0951	$C_{16}H_{18}O_9$	13.5±2.3
**85**	Caffeic acid	180.0423	$C_9H_8O_4$	43.2±11.8
**86**	Butanedioc acid	118.0266	$C_4H_6O_4$	118.3±34.0
**87**	Phenylalanine	165.0790	$C_9H_{11}NO_2$	256.5±28.6
**88**	Uridine	244.0695	$C_9H_{12}N_2O_6$	87.3±13.6
**89**	Cytidine	243.0855	$C_9H_{13}N_3O_5$	1.1±0.1

© 2023 Liu et al. *JAMA Internal Medicine.*

**90**	Adenosine	267.0968	$C_{10}H_{13}N_5O_4$	35.5±17.1
**91**	Guanosine	283.0917	$C_{10}H_{13}N_5O_5$	58.5±10.3
**92**	Adenine	135.0545	$C_5H_5N_5$	23.6±4.6
**93**	Thymine	126.0429	$C_5H_6N_2O_2$	3.3±1.4
**94**	Uracil	112.0273	$C_4H_4N_2O_2$	48.6±9.8
**95**	benzoic acid	122.0368	$C_7H_6O_2$	767.0±133.9
**96**	gallic acid	170.0215	$C_7H_6O_5$	41.1±15.8
**97**	1'-*O*-benzoylsucrose	446.1424	$C_{19}H_{26}O_{12}$	53.0±24.1
**98**	1'-*O*-galloylsucrose	494.1272	$C_{19}H_{26}O_{15}$	3.2±1.6
**99**	6'-*O*-galloylsucrose	494.1272	$C_{19}H_{26}O_{15}$	3.0±1.6
**100**	Trigalloyl glucose	636.0963	$C_{27}H_{24}O_{18}$	42±17.2
**101**	tetragalloyl glucose	788.1072	$C_{34}H_{28}O_{22}$	8.3±5.1
**102**	tetragalloyl glucose isomer	788.1072	$C_{34}H_{28}O_{22}$	2.5±0.8
**103**	(+)-catechin	290.0790	$C_{15}H_{14}O_6$	1.9±2.1
**104**	Ferulic acid	194.0579	$C_{10}H_{10}O_4$	117.0±24.4

Xuebijing is prepared from a combination of *Carthamus tinctorius* flowers (Honghua in Chinese), *Paeonia lactiflora* roots (Chishao), *Ligusticum chuanxiong* rhizomes (Chuanxiong), *Angelica sinensis* roots (Danggui), and *Salvia miltiorrhiza* roots (Danshen).

Analysis of chemical composition of Xuebijing was based on liquid chromatography-mass spectrometry by Professor Chuan Li's laboratory at Shanghai Institute of Materia Medica, Chinese Academy of Sciences (Shanghai, China). A part of these results has been published, while details of the others are pending publication elsewhere.

© 2023 Liu et al. *JAMA Internal Medicine.*

References

(1) Cheng C, Lin JZ, Li L, et al. Pharmacokinetics and disposition of monoterpene glycosides derived from Paeonia lactiflora roots (Chishao) after intravenous dosing of antiseptic Xuebijing injection in human subjects and rats. Acta Pharmacol. Sin. 2016, 37(4): 530–544.

(2) Li XX, Cheng C, Wang FQ, et al. Pharmacokinetics of catechols in human subjects intravenously receiving Xuebijing injection, an emerging antiseptic herbal medicine. Drug Metab. Pharmacokinet. 2016, 31(1): 95–98.

(3) Zhang NT, Cheng C, Olaleye OE, et al. Pharmacokinetics-based identification of potential therapeutic phthalides from XueBiJing, a Chinese herbal injection used in sepsis management. Drug Metab. Dispos. 2018, 46(6): 823–834.

© 2023 Liu et al. *JAMA Internal Medicine.*

The action mechanism of Xuebijing injection

XueBiJing, an intravenous preparation approved by the China Food and Drug Administration (China FDA) in 2004, has been incorporated into routine sepsis care in China. Xuebijing is prepared from a combination of Carthamustinctorius flowers (Honghua in Chinese), Paeonialactiflora roots (Chishao), Ligusticum chuanxiong rhizomes (Chuanxiong), Angelica sinensis roots (Danggui), and Salvia miltiorrhiza roots (Danshen).

Pharmacological studies have shown that Xuebijing has an antagonistic effect on endotoxin, and an inhibitory effect on the uncontrolled release of endogenous inflammatory mediators produced by endotoxin-stimulated monocytes/macrophages. Xuebijing also can improve the coagulation disorder in DIC, increase the activity of superoxide dismutase, regulate hypersensitive or hyposensitive immune response, and prevent the development of organ dysfunction in acute insults. The main pharmacological effects of Xuebijing might be summarized as follows: (1) antagonizing endotoxin; (2) inhibiting inflammatory cytokines; (3) regulating immune function; (4) improving the balance of coagulation; and (5) protecting organ damage.

Xuebijing has pharmacological effects on antagonizing endotoxin, inhibiting inflammatory mediators, improving coagulation function, protecting endothelial cells, improving microcirculation, and regulating immune response. Xuebijing can obviously protect vascular endothelial cells by inhibiting the expression of early/late inflammatory cytokines, attenuate the interaction between inflammation system and coagulation system, and prevent the development of multiple organ dysfunction. Meanwhile, it markedly improves the hyperactive state of the natural immune response during the early stage of sepsis and alleviates the gradually aggravated immunosuppressive state, thereby comprehensively modulating systemic inflammation, coagulopathy and immune dissonance in the development of sepsis. Taken together, these results might suggest the integrated

© 2023 Liu et al. *JAMA Internal Medicine.*

regulation of traditional Chinese medicine on multi-components, multi-pathways, and multi-targets.

Markedly down-regulating synthesis and release of high mobility group box-1 protein (HMGB1), attenuating multi-organ pathological damage, and reducing the mortality rates of experimental animals [1]. Significantly promoting M2 polarization of macrophages, preventing the occurrence and development of MODS, and improving the survival rates of septic mice [2]. Enhancing the apoptosis of regulatory T cells and down-regulating inhibitory function of regulatory T cells on effector T cells [3]. Inhibiting the expression of PAR-1, decreasing the secretion of inflammatory cytokines, and improving coagulation dysfunction in sepsis [4]. Blocking the IRE1α-XBP1 signaling pathway, down-regulating the expression of PDI, and further affecting tissue factor procoagulation activity of endothelial cells [5].

© 2023 Liu et al. *JAMA Internal Medicine.*

References

1. Yao YM, Sheng ZY, Huang LF. The effect of a novel cytokine, high mobility group box-1 protein, on the development of traumatic sepsis. Chin J Integr Med 2009; 15: 13-15.

2. Liu YC, Yao FH, Chai YF, et al. Xuebijing injection promotes M2 polarization of macrophages and improves survival rate in septic mice. Evid-Based Compl Alt Med 2015; 2015: 352642.

3. Ai YH, Yao YM, Dai XG. Effect of apoptosis of CD4+CD25+ regulatory T cells on proliferation as well as secertion of effector T cells and potential influence of Xuebijinginjectiong in septic rats. Chin J Surg 2009; 47: 58-61.

4. Gui YG, Yao YM, Chai YF. A comparison study of interference effect between Xuebijing injection and activated protein C on lipopolysaccharide-induced monocyte tissue factor in rats. Chin J TCM WM Crit Care, 2009, 16(6): 326-329.

5. Chai Y, Li JY, Chai YF, et al. The effect of Xuebijing injection on the procoagulation of tissue factor by inositol-requiring enzyme 1α signaling pathway. Chin J Emerg Med, 2018, 27(2): 159-163.

© 2023 Liu et al. *JAMA Internal Medicine.*

eFigure 1. Tipping Point Analysis for the Primary Outcome

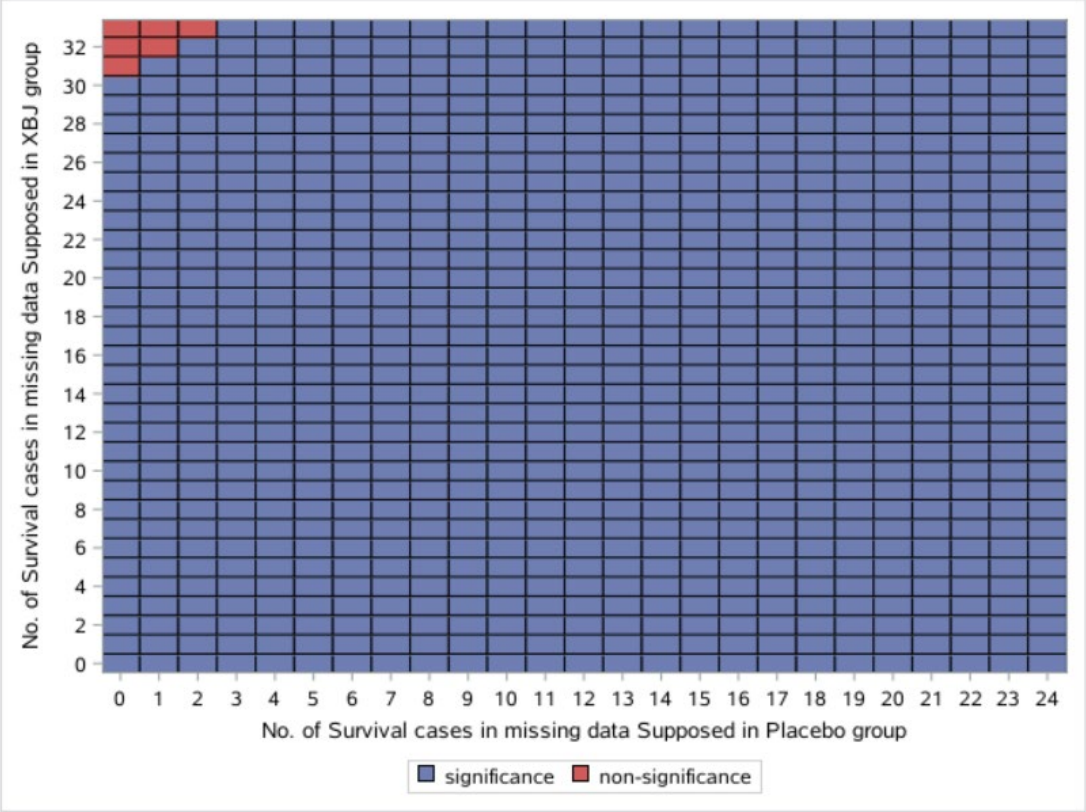

Tipping point analysis method to deal with missing data is to list all possible results caused by missing values by enumeration, and test hypotheses one by one to find the combination that makes the conclusion change, so as to obtain the most comprehensive result in the presence of missing data. Proportion of positive scenarios (POPS, that is, the proportion of combinations of $P<0.05$ to all combinations) often used to reflect the reliability of the success of the test, the larger the POPS value, the greater the reliability of the success of the test. In this study, the proportion of missing data in the experimental group was 3.62% (33/911), and in the control group was 2.65% (24/906). The results obtained by the tipping point analysis were as shown above, because the primary outcome of the trial was a high-quality index (survival rate), the lower right side of the boundary represented "trial success", and the final POPS was 99.27% obtained by TPA analysis in the above figure, indicating that the reliability of the experimental group was better than the control group.

© 2023 Liu et al. *JAMA Internal Medicine.*

eFigure 2. Subgroup Analysis of the Primary Outcome

Subgroup	Placebo group Total No.	Died at Day 28, No.	XBJ group Total No.	Died at Day 28, No.	Difference of mortality %(95% CI)	P Value
All patients						
Overall	882	230	878	165	7.3 (3.4 to 11.2)	<0.001
Age						
≥60	450	135	440	103	6.6 (0.8 to 12.3)	0.008
＜60	432	95	438	62	7.8 (2.7 to 12.9)	0.03
SOFA						
8-13	396	142	392	114	6.8 (0.3 to 13.3)	0.04
2-7	486	88	486	51	7.6 (3.2 to 12.0)	<0.001
APACHE II score						
≥25	29	14	31	16	-3.3 (-28.6 to 22.0)	0.8
＜25	840	206	830	135	8.3 (4.4 to 12.1)	<0.001
MV at baseline						
Yes	483	159	463	121	6.8 (1.0 to 12.6)	0.02
No	392	69	409	44	6.8 (2.0 to 11.7)	0.005
Septic shock						
Yes	412	123	410	96	6.4 (0.4 to 12.5)	0.04
No	457	97	451	55	9.0 (4.2 to 13.8)	<0.001
CRRT						
Yes	92	33	78	22	7.7 (-6.3 to 21.7)	0.28
No	777	187	783	129	7.6 (3.6 to 11.6)	<0.001
ICU types						
General ICU	649	179	664	139	6.6 (2.0 to 11.3)	0.005
Emergency ICU	196	44	185	22	10.6 (3.1 to 18.0)	0.006
Surgical ICU	28	6	16	3	2.7 (-21.8 to 27.1)	0.83
Respiratory ICU	9	1	13	1	3.4 (-21.7 to 28.6)	0.79

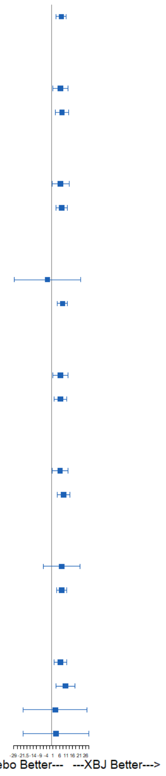

The forest plot shows the difference for the primary outcome (the rate of death from any cause at 28 days) in six prespecified subgroups.

© 2023 Liu et al. *JAMA Internal Medicine.*

eTable 1. Use of Other Medications for Sepsis During Study, N(%)[a]

	Placebo group (N=878)	XBJ group (N=872)
Glucocorticoid	245 (27.9)	221 (25.3)
Anticoagulant	394 (44.9)	411 (47.1)
Vasopressors	395 (45.0)	388 (44.5)
Antimicrobials		
Antibacterial agents	875 (99.7)	871 (99.9)
Carbapenems	512 (58.3)	514 (58.9)
Other beta-lactams	617 (70.3)	581 (66.6)
Quinolones	232 (26.4)	243 (27.9)
Glycopeptide	132 (15.0)	122 (14.0)
Oxazolidinones	165 (18.8)	161 (18.5)
Glycylcyclones	175 (19.9)	173 (19.8)
Aminoglycoside	62 (7.1)	50 (5.7)
Macrolide	13 (1.5)	17 (1.9)
Other[b]	180 (20.5)	180 (20.6)
Antifungal agents	220 (25.1)	207 (23.7)
Antivirals	86 (9.8)	77 (8.8)

[a] Medications used during study was only collected for patients receiving the study drug in the placebo group (n=878) and in the XBJ group (n=872).

[b] Other antibacterial agents included Nitroimidazoles, Sulfanilamide, Polypeptides, Cyclic lipopeptide, Tetracyclines Fosfomycin, Polypeptides, Rifamycin, Anti Mycobacterium drugs, Lincomamides, Chloramphenicols, Furan, Anti tuberculosis drugs, and Rifamycin derivatives.

© 2023 Liu et al. *JAMA Internal Medicine.*

eTable 2. Sensitivity Analyses for the Primary Outcome

28-day mortality	Placebo group (N=906)	XBJ group (N=911)	Difference (95%CI)	*P* Value
Model 1*	26.1(23.2 to 29.0)	18.8(16.2 to 21.4)	7.3(3.4 to 11.2)	<0.001
Model 2	26.1(23.2 to 29.0)	19.1(16.4 to 21.7)	7.0(3.1 to 10.9)	<0.001

* A pre-hoc sensitivity analysis

The analysis of the primary outcome was repeated using 2 analytical approaches.

First, we had 57 cases with missing data. Multiple imputation was used to impute missing values under the missing-at random assumption (MAR). Specifically, 100 imputed data sets were generated using the fully conditional specification method with the number of iterations set to 10 for the following variables: group (XBJ and Placebo), and response variable (28-day mortality: yes, no). After multiple imputation, each of the hundred multiple imputation datasets was analyzed by generalized linear model. The overall estimates were calculated using Rubin's rules. The multiple imputation procedure (PROC MI) in SAS, version 9.4 was used (eTable 2).

Second, a control-based pattern imputation model was assessed whether the primary outcome was robust to departure from MAR. More specifically, an imputation model for the missing observations in the XBJ group was constructed from the observed data in the Placebo group rather than the XBJ group. Parallel to the primary analysis based on MAR, we used a similar method with such an imputed data set to show the robustness of the final results. SAS PROC MI with the MNAR statement was used. The results were robust to departure from MAR (eTable 2).

© 2023 Liu et al. *JAMA Internal Medicine.*

eTable 3. Worse-Case Analysis for the Primary Outcome

eTable3.1. Assume that the missing cases were all deaths

	XBJ group	Placebo group
Death n(%)	198(21.73%)	254(28.04%)
Survival n(%)	713(78.27%)	652(71.96%)
Sum	911	906
The survival rate difference between groups (95%CI)	6.30% (1.17%,10.84%)	
Statistic	3.114	
P value	<0.001	

eTable3.2. It is assumed that the number of missing cases in the XBJ group was dead, and the number of missing cases in the control group was survived

	XBJ group	Placebo group
Death n(%)	198(21.73%)	230(25.39%)
Survival n(%)	713(78.27%)	676(74.61%)
Sum	911	906
The survival rate difference between groups (95%CI)	3.65%(-0.25%,7.55%)	
Statistic	3.364	
P value	0.067	

© 2023 Liu et al. *JAMA Internal Medicine.*

eTable 4. Overall Summary of Adverse Events in Safety Population (N=1750)

	Placebo group (N=878)	XBJ group (N=872)
**Any AE**	222 (25.3)	200 (22.9)
**Investigations**	215 (24.5)	195 (22.4)
ALT increased	42 (4.8)	51 (5.8)
Glutamic-oxaloacetic transaminase	36 (4.1)	38 (4.4)
White blood cell counts increased	38 (4.3)	36 (4.1)
Hemoglobin decreased	31 (3.5)	35 (4.0)
Platelet count decreased	27 (3.1)	24 (2.8)
BUN increased	30 (3.4)	18 (2.1)
Bilirubin total increased	26 (3.0)	16 (1.8)
APTT prolonged	19 (2.2)	17 (1.9)
Fibrinogen increased	17 (1.9)	7 (0.8)
Platelet count increased	9 (1.0)	14 (1.6)
Urine white blood cell increased	8 (0.9)	15 (1.7)
Prothrombin time inc	13 (1.5)	9 (1.0)
Urine red blood cells increased	11 (1.3)	8 (0.9)
Blood creatinine increased	11 (1.3)	6 (0.7)
Sugar blood increased	7 (0.8)	8 (0.9)
Blood creatinine decreased	6 (0.7)	7 (0.8)
Fibrin D dimer increased	5 (0.6)	8 (0.9)
Urinary protein increased	6 (0.7)	7 (0.8)
Decreased white cell count	4 (0.5)	6 (0.7)
Glucose urine elevated	7 (0.8)	3 (0.3)
Fibrinogen decreased	6 (0.7)	3 (0.3)
Faecal occult blood positive	3 (0.3)	4 (0.5)
BUN decreased	1 (0.1)	3 (0.3)
AST decreased	2 (0.2)	0
Blood pressure decreased	2 (0.2)	0
Blood pressure increased	0	2 (0.2)
ALT decreased	1 (0.1)	0
Haemoglobin increased	0	1 (0.1)
**Clinical disorders**	7 (0.8)	5 (0.6)

© 2023 Liu et al. *JAMA Internal Medicine.*

Fever	6 (0.7)	4 (0.5)
Chest tightness	0	1 (0.1)
Pain	1 (0.1)	0
**Gastrointestinal disorders**	1 (0.1)	1 (0.1)
Dry mouth	1 (0.1)	0
Emesis	0	1 (0.1)
**Musculoskeletal and connective tissue disorders**	1 (0.1)	1 (0.1)
Muscle twitching	1 (0.1)	1 (0.1)
**Respiratory, thoracic and mediastinal disorders**	1 (0.1)	1 (0.1)
Breathing difficult	1 (0.1)	1 (0.1)
**Skin and subcutaneous tissue disorders**	1 (0.1)	1 (0.1)
Blistering	1 (0.1)	0
Exanthema	0	1 (0.1)
**Eye disorders**	0	1 (0.1)
Proptosis	0	1 (0.1)
**Psychiatric disorders**	0	1 (0.1)
Restless	0	1 (0.1)
**Any SAE**	20 (2.3)	13 (1.5)
**Cardiac disorders**	13 (1.5)	9 (1.0)
Atrial fibrillation	7 (0.8)	6 (0.7)
Myocardial Infarction	3 (0.3)	1 (0.1)
Arrhythmic storm	2 (0.2)	1 (0.1)
Arrhythmia ventricular	1 (0.1)	1 (0.1)
**Gastrointestinal disorders**	5 (0.6)	2 (0.2)
Upper gastrointestinal hemorrhage	5 (0.6)	2 (0.2)
**Nervous system disorders**	2 (0.2)	2 (0.2)
Brain infarction	2 (0.2)	2 (0.2)
**Blood and lymphatic system disorders**	1 (0.1)	0
Disseminated intravascular coagulation	1 (0.1)	0

© 2023 Liu et al. *JAMA Internal Medicine.*
