# Supplementary material for: Effect of an Herbal-Based Injection on 28-Day Mortality in Patients With Sepsis: The EXIT-SEP Randomized Clinical Trial
Source: JAMA Intern Med. 2023 May 1;183(7):647–55. doi: 10.1001/jamainternmed.2023.0780 (PMC10152378; doi:10.1001/jamainternmed.2023.0780)
Supplement: Supplement 3. — Nonauthor Collaborators. EXIT-SEP Investigators [file jamainternmed-e230780-s003.pdf]

Supplemental Online Content: Nonauthor Collaborators
*First name, last name, and suffix (if applicable) are required and will appear in PubMed.

*Group Name(s): EXIT-SEP Investigators							
***First Name and Middle Initial(s)**	***Last Name**	***Suffix (eg, Jr, III)**	Academic Degrees	Institution	Location (city, state/province, country)	Role or Contribution, eg, chair, principal investigator	Group (if more than 1 Group listed in the byline) and/or Subgroup (eg, Steering Committee)
Wenxue	Liu		M.D.	Department of Critical Care Medicine, Zhongda Hospital, School of Medicine, Southeast University	Nanjing, Jiangsu, China	Data collection	
Meixia	Shang		Ph.D.	Department of Medical Statistics, Peking University First Hospital	Beijing, China	Data management	
Jibin	Han		M.D.	Department of Critical Care Medicine, The First Hospital of Shanxi Medical University	Taiyuan, Shanxi, China	Data collection	
Ming	Li		M.D.	Department of Critical Care Medicine, The Second Affiliated Hospital of Harbin Medical University	Harbin, Heilongjiang, China	Data collection	
Linhao	Ma		M.D.	Department of Emergency, Shanghai Changzheng Hospital	Shanghai, China	Data collection	
Yunpeng	Li		M.D.	Department of Critical Care Medicine, Henan Provincial People's Hospital	Zhengzhou, Henan, China	Data collection	
Yuanyi	Liu		M.D.	Department of Critical Care Medicine, The Affiliated Hospital of Guizhou Medical University	Guiyang, Guizhou, China	Data collection	
Tao	Yu		M.D.	Department of Critical Care Medicine, Yijishan Hospital, The First Affiliated Hospital of Wannan Medical College, Wuhu, Anhui, China	Wuhu, Anhui, China	Data collection	
Xin	Li		M.D.	Department of Critical Care Medicine, The First Affiliated Hospital, China Medical University	Shenyang, Liaoning, China	Data collection	

Supplemental Online Content: Nonauthor Collaborators
*First name, last name, and suffix (if applicable) are required and will appear in PubMed.

***First Name and Middle Initial(s)**	***Last Name**	***Suffix (eg, Jr, III)**	Academic Degrees	Institution	Location (city, state/province, country)	Role or Contribution, eg, chair, principal investigator	Group (if more than 1 Group listed in the byline) and/or Subgroup (eg, Steering Committee)
Xing	Lu		M.D.	Department of Critical Care Medicine, Tianjin Third Central Hospital	Tianjin, China	Data collection	
Qihong	Chen		M.D.	Department of Critical Care Medicine, Northern Jiangsu People's Hospital	Yangzhou, Jiangsu, China	Data collection	
Yimin	Yang		M.D.	Department of Critical Care Medicine, The First Hospital of Jilin University	Changchun, Jilin, China	Data collection	
Yan	Li		M.D.	Department of Emergency, China-Japan Friendship Hospital	Beijing, China	Data collection	
Weiguang	Guo		M.D.	Department of Critical Care Medicine, First People's Hospital of Foshan	Foshan, Guangdong, China	Data collection	
Zhimin	Dou		M.D.	Department of Critical Care Medicine, The First Hospital of Lanzhou University	Lanzhou, Gansu, China	Data collection	
Chengjin	Gao		M.D.	Department of Emergency, Xinhua Hospital, School of Medicine, Shanghai Jiaotong University	Shanghai, China	Data collection	
Dahuan	Li		M.D.	Department of Emergency, The First Affiliated Hospital of Henan Science and Technology University	Luoyang, Henan, China	Data collection	
Xiaotong	Han		M.D.	Department of Emergency, Hunan Provincial People's Hospital	Changsha, Hunan, China	Data collection	
Qiang	Shao		M.D.	Department of Critical Care Medicine, The First Affiliated Hospital of Nanchang University	Nanchang, Jiangxi, China	Data collection	

Supplemental Online Content: Nonauthor Collaborators
*First name, last name, and suffix (if applicable) are required and will appear in PubMed.

***First Name and Middle Initial(s)**	***Last Name**	***Suffix (eg, Jr, III)**	Academic Degrees	Institution	Location (city, state/province, country)	Role or Contribution, eg, chair, principal investigator	Group (if more than 1 Group listed in the byline) and/or Subgroup (eg, Steering Committee)
Yun	Xie		M.D.	Department of Emergency, Shanghai General Hospital, School of Medicine, Shanghai Jiaotong University	Shanghai, China	Data collection	
Xiaoli	Li		M.D.	Department of Critical Care Medicine, Yantai Yuhuangding Hospital of Qingdao University	Yantai, Shandong, China	Data collection	
Jin	Lin		M.D.	Department of Critical Care Medicine, Beijing Friendship Hospital, Capital Medical University	Beijing, China	Data collection	
Zhi	Li		M.D.	Department of Critical Care Medicine, Qilu Hospital of Shandong University (Qingdao)	Qingdao, Shandong, China	Data collection	
Min	Gao		M.D.	Department of Critical Care Medicine, The First Affiliated Hospital of Zhengzhou University	Zhengzhou, Henan, China	Data collection	
Yunxi	Song		M.D.	Department of Respiration, Chinese PLA General Hospital of Rocket Forces	Beijing, China	Data collection	
Binxiao	Su		M.D.	Department of Anesthesiology, Xijing Hospital	Xi'an, Shaanxi, China	Data collection	
Yun	Liu		M.D.	Department of Critical Care Medicine, Jiangsu Province Hospital	Nanjing, Jiangsu, China	Data collection	
Yue	Peng		M.D.	Department of Critical Care Medicine, The Third Xiangya Hospital of Central South University	Changsha, Hunan, China	Data collection	

*First name, last name, and suffix (if applicable) are required and will appear in PubMed.

***First Name and Middle Initial(s)**	***Last Name**	***Suffix (eg, Jr, III)**	Academic Degrees	Institution	Location (city, state/province, country)	Role or Contribution, eg, chair, principal investigator	Group (if more than 1 Group listed in the byline) and/or Subgroup (eg, Steering Committee)
Qingsong	Cui		M.D.	Department of Critical Care Medicine, Yanbian University Hospital	Yanji, Jilin, China	Data collection	
Huichao	Yu		M.D.	Department of Critical Care Medicine, Central Hospital of Shenyang Medical College	Shenyang, Liaoning, China	Data collection	
Baocai	Fu		M.D.	Department of Critical Care Medicine, Yantaishan Hospital	Yantai, Shandong, China	Data collection	
Shihui	Lin		M.D.	Department of Critical Care Medicine, The First Affiliated Hospital of Chongqing Medical University	Chongqing, China	Data collection	
Yanxia	Huang		M.D.	Department of Critical Care Medicine, Ruijin North Hospital, School of Medicine, Shanghai Jiaotong University	Shanghai, China	Data collection	
Xuedan	Cao		M.D.	Department of Critical Care Medicine, First Affiliated Hospital, Heilongjiang University of Chinese Medicine	Harbin, Heilongjiang, China	Data collection	
Xiao	Wang		M.D.	Department of Emergency, First Affiliated Hospital of Kunming Medical University	Kunming, Yunnan, China	Data collection	
Qing	Fang		M.D.	Department of Emergency, Chinese PLA Wuhan General Hospital	Wuhan, Hubei, China	Data collection	
Ruolan	Huang		M.D.	Department of Critical Care Medicine, Shenzhen Traditional Chinese Medicine Hospital	Shenzhen, Guangdong, China	Data collection	
Kaichao	Yang		M.D.	Department of Emergency, Shanghai Sixth People's Hospital	Shanghai, China	Data collection	

Supplemental Online Content: Nonauthor Collaborators
*First name, last name, and suffix (if applicable) are required and will appear in PubMed.

***First Name and Middle Initial(s)**	***Last Name**	***Suffix (eg, Jr, III)**	Academic Degrees	Institution	Location (city, state/province, country)	Role or Contribution, eg, chair, principal investigator	Group (if more than 1 Group listed in the byline) and/or Subgroup (eg, Steering Committee)
Ying	Feng		M.D.	Department of Critical Care Medicine, Zhongnan Hospital of Wuhan University	Wuhan, Hubei, China	Data collection	
Fangxiao	Gong		M.D.	Department of Critical Care Medicine, Zhejiang Provincial People's Hospital	Hangzhou, Zhejiang, China	Data collection	
Jun	Yin		M.D.	Department of Emergency, Zhongshan Hospital, Fudan University	Shanghai, China	Data collection	
Shixia	Cai		M.D.	Department of Critical Care Medicine, The Affiliated Hospital of Qingdao University	Qingdao, Shandong, China	Data collection	
Shixin	Li		M.D.	Department of Emergency, Tianjin Medical University General Hospital	Tianjin, China	Data collection	
Jingchao	Wang		M.D.	Department of Critical Care Medicine, The Affiliated Hospital of Inner Mongolia Medical University	Hohhot, Inner Mongolia, China	Data collection	
Wenke	Zheng		Ph.D.	Evidence-Based Medicine Center, Tianjin University of Traditional Chinese Medicine	Tianjin, China	Quality Control	
