# Supplementary material for: Effect of an Herbal-Based Injection on 28-Day Mortality in Patients With Sepsis: The EXIT-SEP Randomized Clinical Trial
Source: JAMA Intern Med. 2023 May 1;183(7):647–55. doi: 10.1001/jamainternmed.2023.0780 (PMC10152378; doi:10.1001/jamainternmed.2023.0780)
Supplement: Supplement 4. — Data Sharing Statement [file jamainternmed-e230780-s004.pdf]

Data Sharing Statement

Liu. Effect of an Herbal-Based Injection on 28-Day Mortality in Patients With Sepsis. *JAMA Intern Med*. Published May 01, 2023. doi:10.1001/jamainternmed.2023.0780

Data
Data available: Yes
Data types: Deidentified participant data, Data dictionary
How to access data: Data collected for the study, including deidentified participant data and a data dictionary defining each field in the set, will be made available to other investigators upon request to the corresponding author, after approval of a proposal by the EXIT-SEP Investigators boards, with a signed data access agreement.
When available: With publication

Supporting Documents
Document types: None

Additional Information
Who can access the data: The trial protocol will be made available to other investigators with publication.
Types of analyses: The trial protocol.
Mechanisms of data availability: the data will be made available after manuscript is accepted for publication, without investigator support.
